# Bisavenathramide Analogues as Nrf2 Inductors and Neuroprotectors in In Vitro Models of Oxidative Stress and Hyperphosphorylation

**DOI:** 10.3390/antiox10060941

**Published:** 2021-06-10

**Authors:** Ángel Cores, Sheila Abril, Patrycja Michalska, Pablo Duarte, Ana I. Olives, M. Antonia Martín, Mercedes Villacampa, Rafael León, J. Carlos Menéndez

**Affiliations:** 1 Unidad de Química Orgánica y Farmacéutica, Departamento de Química en Ciencias Farmacéuticas, Facultad de Farmacia, Universidad Complutense, 28040 Madrid, Spain; acores@ucm.es (Á.C.); mvsanz@ucm.es (M.V.); 2 Instituto Teófilo Hernando y Departamento de Farmacología y Terapéutica, Facultad de Medicina, Universidad Autónoma de Madrid, 28029 Madrid, Spain; sheisheiabril@gmail.com (S.A.); patrycjamdziama@gmail.com (P.M.); pablo.duarte@uam.es (P.D.); 3 Instituto de Química Médica, Consejo Superior de Investigaciones Científicas (IQM-CSIC), 28006 Madrid, Spain; 4 Unidad de Química Analítica, Departamento de Química en Ciencias Farmacéuticas, Facultad de Farmacia, Universidad Complutense, 28040 Madrid, Spain; aiolives@ucm.es (A.I.O.); mantonia@farm.ucm.es (M.A.M.)

**Keywords:** Nrf2–ARE pathway, neurodegenerative diseases, oxidative stress, Keap1, Nrf2 regulation, bisavenanthramides

## Abstract

Oxidative stress is crucial to the outbreak and advancement of neurodegenerative diseases and is a common factor to many of them. We describe the synthesis of a library of derivatives of the 4-arylmethylen-2-pyrrolin-5-one framework by sequential application of a three-component reaction of primary amines, β-dicarbonyl compounds, and α-haloketones and a Knoevenagel condensation. These compounds can be viewed as cyclic amides of caffeic and ferulic acids, and are also structurally related to the bisavenanthramide family of natural antioxidants. Most members of the library showed low cytotoxicity and good activity as inductors of Nrf2, a transcription factor that acts as the master regulator of the antioxidant response associated with activation of the antioxidant response element (ARE). Nrf2-dependent protein expression was also proved by the significant increase in the levels of the HMOX1 and NQO1 proteins. Some compounds exerted neuroprotective properties in oxidative stress situations, such as rotenone/oligomycin-induced toxicity, and also against protein hyperphosphorylation induced by the phosphatase inhibitor okadaic acid. Compound **3i**, which can be considered a good candidate for further hit-to-lead development against neurodegenerative diseases due to its well-balanced multitarget profile, was further characterized by proving its ability to reduce phosphorylated Tau levels.

## 1. Introduction

Reactive oxygen species (ROS) have important physiological roles, since they act as second messengers between cells at moderate concentrations. However, oxidative stress, i.e., an imbalance between the production of ROS and the ability of antioxidant defenses to neutralize them, causes brain tissue damage and neuronal death [1,2,3] and is one of the main hallmarks in neurodegeneration. 

Cells have developed a number of antioxidant molecules and detoxifying enzymes as defenses against oxidative stress. One of them is the Nrf2 protein (nuclear factor erythroid 2-related factor 2), which acts as the master regulator of cellular redox homeostasis. For this reason, Nrf2 activation is an effective mechanism against oxidative or electrophilic stress [4], and Nrf2-connected pathways are increasingly important targets in drug discovery in many therapeutic areas, and, in particular, in neurodegeneration [5].

Under physiological conditions, Nrf2 is in the cytosol, bound to its negative regulator Keap1, which eventually facilitates Nrf2 ubiquitination by an E3 ligase and its proteasomal degradation [6]. As a consequence of oxidative stress, cysteine residues in the sensor region of Keap1 are oxidized to cystine, a transformation that induces a conformational change of the protein and the subsequent loss of its affinity for Nrf2. A similar behavior is observed upon Cys alkylation by electrophiles. After its release from Keap1, Nrf2 can be translocated into the nucleus and, after recruiting several coactivator proteins, it binds to ARE (antioxidant response element) sequences [4], promoting the transcription of several cytoprotective genes. These Nrf2-target genes codify the synthesis of enzymes able to scavenge ROS and neutralize electrophiles, including heme-oxygenase-1 (HO-1), NAD(P)H:quinone oxidoreductase 1 (NQO1), superoxide dismutase (SOD), catalase, glutathione reductase (GR), glutathione peroxidase, and glutamate cysteine ligase (GCL) [7]. 

Some natural products having an α,β-unsaturated carbonyl moiety have received much attention as Nrf2 inducers [8]. They include caffeic acid esters, which show anti-inflammatory and immunomodulatory properties [9], and ferulic acid and its derivatives, which efficiently protect proteins and lipids against oxidation [10]. Avenanthramides (AVAs) [11] are amides from anthranilic acid and antioxidant cinnamic acid derivatives, such as caffeic, ferulic, or *p*-coumaric acids [11]. In vitro and in vivo studies have shown that AVAs display potent antioxidant capacity in a variety of antioxidant assays, and they are also able to scavenge several other biologically relevant radical species and induce the biosynthesis of cytoprotective proteins. Besides radical-scavenging hydroxyl and amide groups, AVAs display an α,β-unsaturated amide group, which acts as a Michael acceptor for cysteine residues within the sensor region of Keap1, promoting the production of cytoprotective enzymes mediated by Nrf2 [12,13]. 

**Figure 1:**
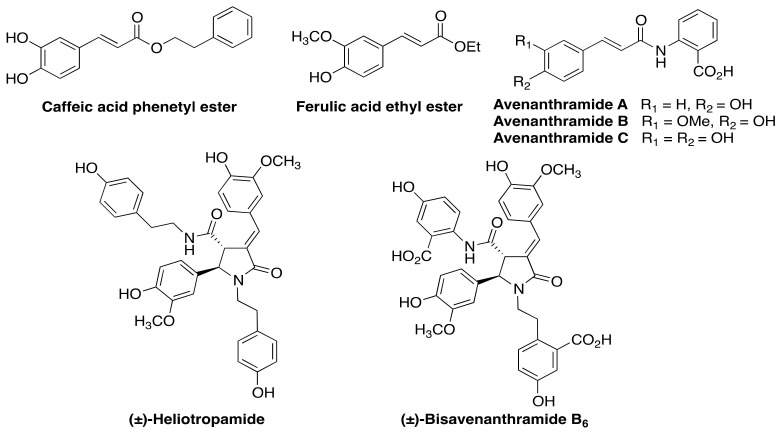
Some natural cinnamic acid derivatives with antioxidant properties.

Upon exposure to reactive oxygen species, avenanthramides are transformed into cyclic compounds based on a dimeric 2-pyrrolinone core, such as heliotropamide A and bisavenanthramide B-6 (Figure 1). These compounds retain the antioxidant activity and show some new properties, including acetylcholinesterase inhibitory activity [11], and they may, therefore, be regarded as promising against neurodegenerative disorders. 

**Figure 2:**
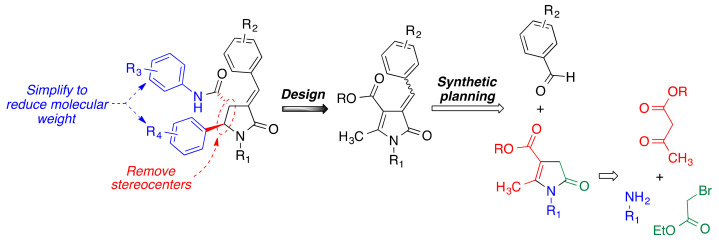
Compound design and synthetic planning used in this article.

In spite of their good pharmacological profiles, bisavenathramides have several disadvantages from a drug discovery perspective. In the first place, their drug-likeness is hampered by their high molecular weights. Their chirality also poses potential problems, and, indeed, naturally occurring bisavenathramides are a mixture of both enantiomers due to their racemization under radical conditions [14]. These shortcomings inspired us to design a new family of simplified 2-pyrrolinones, avoiding chirality and reducing molecular weight, while maintaining the critical α,β-unsaturated lactam and phenol functions [15]. For the preparation of the proposed pyrrolinone core, we planned to take advantage of our recently developed three-component reaction between primary amines, α-haloesters, and β-dicarbonyl compounds [16], which would be followed by a Knoevenagel condensation to install the α,β-insaturation (Figure 2). We expected these compounds to display a similar profile to the natural models and, in particular, we were interested in studying them as inductors of Nrf2, the master regulator of the Phase II anti-oxidative response [17] and an emerging target in drug discovery in the neurodegeneration area [5]. While avenanthramides have been well characterized as Nrf2 inducers, a feature that was ascribed to their α,β-unsaturated amide moiety [12,13], bisavenanthramides have not been studied in this regard although they show the same structural feature. 

## 2. Materials and Methods 

### 2.1. Chemistry: General Information 

All commercial reagents and solvents were used as received. Reactions were monitored by thin layer chromatography on silica-gel-coated aluminium plates containing a fluorescent indicator. Microwave-assisted reactions were performed on a CEM Discover focused microwave reactor. Separations by flash chromatography were performed on conventional silica gel columns or on a Combiflash Teledyne automated flash chromatograph. Melting points were measured with a Kofler-type heating platine microscope from Reichert, model 723, and are uncorrected. Infrared spectra were obtained with an Agilent Cary630 FTIR spectrophotometer with a diamond ATR accessory for solid and liquid samples, and wavenumbers are given in cm^−1^. NMR data were obtained using a Bruker Avance spectrometer (NMR Unit, Universidad Complutense, Madrid, Spain), working at 250 MHz for ^1^H NMR and 63 MHz for ^13^C NMR; chemical shifts are given in parts per million (δ scale) and coupling constants (*J*) are given in hertz. Combustion elemental analyses were obtained by the CAI de Microanálisis, Universidad Complutense, using a Leco CHNS-932 combustion microanalyzer. UV–VIS absorption measurements at fixed wavelength were carried out on a 047-10111 microplate reader from Pike Technologies (Madison, WI, USA), assisted by G6866A holder for the optical fiber probe, and coupled to a UV–VIS spectrophotometer Cary 60 from Agilent (Santa Clara, CA, USA). The spectrophotometer is equipped with control and data acquisition Cary WinUV Software. Programming and control of the microplate reader was performed through Pike Technologies AutoPRO software (Madison, WI, USA). Transparent polystyrene plates containing 96 wells were employed. An Accublock D-1301 digital dry bath from Labnet (Madrid, Spain) was employed for incubating the reaction media in the FRAP experiments.

### 2.2. Synthesis of 2-Pyrrolin-5-one Derivatives ***1***

Compounds **1a**–**c** were synthesized by our previously reported method [16], and their spectral data were identical to those already published. 

### 2.3. Synthesis of Non-Commercial 4-benzyloxy-3-methoxybenzaldehydes ***2***

K_2_CO_3_ and KI were added to a solution of vanillin and the suitable benzyl chloride derivative in acetonitrile (10 mL), and the suspension was refluxed while stirred for 3 h. The reaction mixture was allowed to cool to room temperature and, after the addition of ethyl acetate (20 mL), it was washed with brine (2 × 20 mL). The organic layer was dried over anhydrous sodium sulphate and filtered, and the solvent was evaporated in vacuo. The solid residue was triturated with hexane (3 × 10 mL) to furnish the desired aldehydes. 

#### 2.3.1. 4-((2-Fluorobenzyl)oxy)-3-methoxybenzaldehyde (**2a**)

Prepared from vanillin (3 mmol) and 2-fluorobenzyl chloride (3.3 mmol); isolated as a yellow solid (757 mg, 97%); mp: 62–63 °C; ^1^H NMR (250 MHz, CDCl_3_) δ 9.67 (s, 1H), 7.34 (td, *J* = 7.5, 1.7 Hz, 1H), 7.27–7.21 (m, 2H), 7.13 (ddd, *J* = 7.4, 5.5, 2.0 Hz, 1H), 7.05–6.93 (m, 1H), 6.93–6.87 (m, 1H), 6.86 (d, *J* = 8.2 Hz, 1H), 5.13 (s, 2H), 3.77 (s, 3H); ^13^C NMR (63 MHz, CDCl_3_) δ 191.1, 160.4 (d, *J* = 246.9 Hz), 153.4, 150.2, 130.6, 130.1 (d, *J* = 8.2 Hz), 129.6 (d, *J* = 3.8 Hz), 126.8, 124.6 (d, *J* = 3.6 Hz), 123.3 (d, *J* = 14.1 Hz), 115.6 (d, *J* = 21.1 Hz), 112.3, 109.5, 64.6 (d, *J* = 4.6 Hz), 56.2; IR (neat, cm^−1^): 1693.9, 1582.9, 1499.6, 1260.5, 1228.9; elemental analysis (%) calculated for C_15_H_13_FO_3_: C 69.22, H 5.03; found: C 68.99, H 4.89.

#### 2.3.2. 4-((2-Chlorobenzyl)oxy)-3-methoxybenzaldehyde (**2b**)

Prepared from vanillin (3.16 mmol) and 2-chlorobenzyl chloride (3.48 mmol); isolated as a yellow solid (813 mg, 93%); mp: 48 °C; ^1^H NMR (250 MHz, CDCl_3_) δ 9.85 (s, 1H), 7.58–7.49 (m, 1H), 7.47–7.35 (m, 3H), 7.32–7.23 (m, 2H), 6.97 (d, *J* = 8.1 Hz, 1H), 5.34 (s, 2H), 3.97 (s, 3H); ^13^C NMR (63 MHz, CDCl_3_) δ 191.1, 153.3, 150.2, 133.8, 132.4, 130.6, 129.6, 129.4, 128.6, 127.3, 126.8, 112.5, 109.5, 68.0, 56.2; IR (neat, cm^−1^): 1681.4, 1587.5, 1507.6, 1265.2; elemental analysis (%) calculated for C_15_H_13_ClO_3_: C 65.11, H 4.74; found: C 65.27, H 4.61.

#### 2.3.3. 4-((4-Chlorobenzyl)oxy)-3-methoxybenzaldehyde (**2c**)

Prepared from vanillin (1.41 mmol) and 4-chlorobenzyl chloride (1.55 mmol); isolated as a yellow solid (370 mg, 95%); mp: 82 °C; ^1^H NMR (250 MHz, CDCl_3_) δ 9.87 (s, 1H), 7.46 (d, *J* = 1.8 Hz, 1H), 7.45–7.38 (m, 5H), 6.99 (d, *J* = 8.1 Hz, 1H), 5.23 (s, 2H), 3.98 (s, 3H); ^13^C NMR (63 MHz, CDCl_3_) δ 191.1, 153.4, 150.2, 134.6, 134.2, 130.6, 129.1 (2C), 128.7 (2C), 126.7, 112.5, 109.5, 70.2, 56.2; IR (neat, cm^−1^): 1668.3, 1580.4, 1504.2, 1258.3, 1227.2; elemental analysis (%) calculated for C_15_H_13_ClO_3_: C 65.11, H 4.74; found: C 65.22, H 4.68.

### 2.4. General Synthesis of 4-Arylmethylen-2-pyrrolin-5-ones ***3***

To a suspension of the suitable 2-pyrrolin-5-one **1** (0.25 mmol, 1 eq) in ethanol (10 mL) was added the suitable aldehyde (0.28 mmol, 1.1 eq) and piperidine (0.5 mmol, 2 eq). The resulting solution was refluxed while monitored by TLC. Upon completion of the reaction, as judged by the absence of the starting 2-pyrrolin-5-one, the solution was cooled to room temperature. After the addition of ethyl acetate (20 mL), it was washed with water (2 × 10 mL). The organic layer was dried over anhydrous sodium sulphate and filtered, and the solvent was evaporated in vacuo, giving a residue that was purified by chromatography on silica gel, using as the mobile phase a gradient from neat hexane to 1:1 hexane-ethyl acetate.

#### 2.4.1. Methyl (*Z*)-1-Benzyl-4-benzylidene-2-methyl-5-oxo-4,5-dihydro-1*H*-pyrrole-3-carboxylate (**3a**) 

Prepared from pyrrolinone **1a** (1 mmol) and benzaldehyde (1.1 mmol); isolated as a yellow solid (167 mg, 50%); mp: 83–86 °C; ^1^H NMR (250 MHz, CDCl_3_) δ 8.28 (s, 1H), 8.22–8.11 (m, 2H), 7.54–7.14 (m, 8H), 4.90 (s, 2H), 3.87 (s, 3H), 2.45 (s, 3H); ^13^C NMR (63 MHz, CDCl_3_) δ 166.2, 165.2, 153.4, 142.2, 137.1, 135.0, 132.1 (2C), 130.5, 129.3 (2C), 128.4 (2C), 128.0, 127.2 (2C), 126.8, 104.3, 51.4, 43.8, 14.2; IR (neat, cm^−1^): 1684, 1599, 1193; elemental analysis (%) calculated for C_21_H_19_NO_3_: C 75.66, H 5.74, N 4.20; found: C 75.55, H 5.68, N 4.18.

#### 2.4.2. Methyl (*Z*)-1-Benzyl-4-(4-methoxybenzylidene)-2-methyl-5-oxo-4,5-dihydro-1*H*-pyrrole-3-carboxylate (**3b**)

Prepared from pyrrolinone **1a** (1 mmol) and 4-methoxybenzaldehyde (1.1 mmol); isolated as a yellow solid (218 mg, 60%); mp: 121–124 °C; ^1^H NMR (250 MHz, CDCl_3_) δ 8.39–8.08 (m, 3H), 7.47–7.10 (m, 5H), 7.03–6.83 (m, 2H), 4.93 (s, 2H), 3.89 (s, 3H), 3.86 (s, 3H), 2.44 (s, 3H); ^13^C NMR (63 MHz, CDCl_3_) δ 166.1, 165.1, 161.5, 151.3, 142.1, 137.0, 134.5 (2C), 132.4, 129.0 (2C), 127.7, 126.9 (2C), 124.3, 113.6 (2C), 104.2, 55.5, 51.0, 43.5, 13.8; IR (neat, cm^−1^): 1679.0, 1589.4, 1254.4; elemental analysis (%) calculated for C_22_H_21_NO_4_: C 72.71, H 5.82, N 3.85; found: C 72.46, H 5.84, N 3.92.

#### 2.4.3. Methyl (*Z*)-1-Benzyl-4-(4-chlorobenzylidene)-2-methyl-5-oxo-4,5-dihydro-1*H*-pyrrole-3-carboxylate (**3c**) 

Prepared from pyrrolinone **1a** (1 mmol) and 4-chlorobenzaldehyde (1.1 mmol); isolated as a yellow solid (166 mg, 45%); mp: 97–100 °C; ^1^H NMR (250 MHz, CDCl_3_) δ 8.09 (s, 1H), 8.00 (d, *J* = 8.4 Hz, 2H), 7.35–7.20 (m, 5H), 7.16–7.06 (m, 2H), 4.81 (s, 2H), 3.76 (s, 3H), 2.36 (d, *J* = 0.6 Hz, 3H); ^13^C NMR (63 MHz, CDCl_3_) δ 166.2, 165.1, 153.6, 140.6, 136.9, 136.3, 133.4 (2C), 129.4, 129.3 (2C), 128.7 (2C), 128.1, 127.3, 127.1 (2C), 104.2, 51.4, 43.8, 14.2; IR (neat, cm^−1^): 1691, 1677, 1602, 1196; elemental analysis (%) calculated for C_21_H_18_ClNO_3_: C 68.57, H 4.93, N 3.81; found: C 68.20, H 4.88, N 3.81.

#### 2.4.4. Methyl (*Z*)-4-(Benzo[*d*](1,3)dioxol-5-ylmethylene)-2-methyl-5-oxo-1-phenethyl-4,5-dihydro-1*H*-pyrrole-3-carboxylate (**3d**)

Prepared from pyrrolinone **1b** (0.25 mmol) and piperonal (0.28 mmol); isolated as a yellow solid (235 mg, 60%); mp: 179 °C; ^1^H NMR (250 MHz, CDCl_3_) δ 8.17 (d, *J* = 1.6Hz, 1H), 8.13 (s, 1H), 7.56 (dd, *J* = 8.2, 1.6 Hz, 1H), 7.38–7.11 (m, 5H), 6.88 (d, *J* = 8.2 Hz, 1H), 6.05 (s, 2H), 3.87 (t, *J* = 7.3 Hz, 2H), 3.85 (s, 3H), 2.93 (t, *J* = 7.3 Hz, 2H,), 2.25 (s, 3H); ^13^C NMR (63 MHz, CDCl3) δ 166.1, 165.3, 151.9, 149.9, 147.8, 141.9, 138.6, 129.6, 129.3 (2C), 129.1 (2C), 127.2, 125.1, 111.7, 108.3, 103.9, 101.9, 51.3, 42.5, 35.8, 13.4; IR (neat, cm^−1^): 1672.7, 1264.8; elemental analysis (%) calculated for C_23_H_11_NO_5_: C 70.58, H 5.41, N 3.58; found: C 70.38, H 5.32, N 3.36. 

#### 2.4.5. Methyl (*Z*)-4-(2,4-Dimethoxybenzylidene)-2-methyl-5-oxo-1-phenethyl-4,5-dihydro-1*H*-pyrrole-3-carboxylate (**3e**) 

Prepared from pyrrolinone **1b** (0.25 mmol) and 2,4-dimethoxybenzaldehyde (0.28 mmol); isolated as a yellow solid (42 mg, 41%); mp: 126 °C; ^1^H NMR (250 MHz, CDCl_3_) δ 8.48 (d, *J* = 8.8 Hz, 1H), 8.41 (s, 1H), 7.35–7.07 (m, 5H), 6.55 (dd, *J* = 8.8, 2.4 Hz, 1H), 6.44 (d, *J* = 2.4 Hz, 1H), 3.87 (s, 3H), 3.86 (s, 3H), 3.85–3.77 (m, 5H), 2.91–2.82 (m, 2H), 2.22 (s, 3H); ^13^C NMR (63 MHz, CDCl_3_) δ 166.1, 165.2, 163.2, 160.2, 151.3, 138.5, 136.6, 133.3, 129.1 (2C), 128.9 (2C), 126.9, 124.2, 116.8, 104.1, 97.9, 55.9, 55.6, 51.0, 42.2, 35.6, 13.0; IR (neat, cm^−1^): 1680.6, 1594.6, 1268.6, 1205.0; elemental analysis (%) calculated for C_24_H_25_NO_5_: C 70.75, H 6.18, N 3.44; found: C 70.93, H 6.09, N 3.20.

#### 2.4.6. Methyl (*Z*)-2-Methyl-5-oxo-1-phenethyl-4-(2,4,5-trimethoxybenzylidene)-4,5-dihydro-1*H*-pyrrole-3-carboxylate (**3f**) 

Prepared from pyrrolinone **1b** (0.25 mmol) and 2,4,5-trimethoxybenzaldehyde (0.28 mmol); isolated as a yellow solid (51 mg, 47%); mp: 141 °C; ^1^H NMR (250 MHz, CDCl_3_) δ 8.55 (s, 2H), 7.33–7.12 (m, 5H), 6.49 (s, 1H), 3.95 (m, 6H), 3.90 (s, 3H), 3.85–3.79 (m, 5H), 2.89 (t, *J* = 7.3 Hz, 2H), 2.20 (s, 3H); ^13^C NMR (63 MHz, CDCl_3_) δ 166.0, 165.2, 155.0, 152.5, 150.9, 142.2, 138.6, 135.8, 129.1 (2C), 128.8 (2C), 126.9, 123.7, 115.9, 114.8, 104.2, 95.9, 56.8, 56.6, 56.1, 51.0, 42.1, 35.6, 13.0; IR (neat, cm^−1^): 1675.3, 1587.8, 1278.9, 1205.3; elemental analysis (%) calculated for C_25_H_27_NO_6_: C 68.64, H 6.22, N 3.20; found: C 68.45, H 6.19, N 3.31.

#### 2.4.7. Methyl (*Z*)-2-Methyl-5-oxo-1-phenethyl-4-(3,4,5-trimethoxybenzylidene)-4,5-dihydro-1*H*-pyrrole-3-carboxylate (**3g**) 

Prepared from pyrrolinone **1b** (0.25 mmol) and 3,4,5-trimethoxybenzaldehyde (0.28 mmol); isolated as a yellow solid (58 mg, 53%); mp: 170 °C; ^1^H NMR (250 MHz, CDCl_3_) δ 8.15 (s, 1H), 7.64 (s, 2H), 7.35–7.12 (m, 5H), 3.93 (s, 6H), 3.91 (s, 3H), 3.88–3.82 (m, 5H), 2.90 (t, *J* = 7.2 Hz, 2H), 2.20 (s, 3H); ^13^C NMR (63 MHz, CDCl_3_) δ 165.8, 165.0, 152.6 (2C), 152.1, 141.7, 140.1, 138.4, 130.3, 129.1 (2C), 128.8 (2C), 126.9 (2C), 125.9, 109.8, 103.5, 61.1, 56.4 (2C), 51.1, 42.1, 35.5, 13.3; IR (neat, cm^−1^): 3255.4, 1688.5, 1651.8, 1593.2, 1386.6, 1365.7, 1226.7; elemental analysis (%) calculated for C_25_H_27_NO_6_: C 74.59, H 5.74, N 7.25; found: C 73.45, H 5.66, N 7.22.

#### 2.4.8. Methyl (*Z*)-4-(3-Hydroxy-4-methoxybenzylidene)-2-methyl-5-oxo-1-phenethyl-4,5-dihydro-1*H*-pyrrole-3- carboxylate (**3h**) 

Prepared from pyrrolinone **1b** (0.25 mmol) and 3-hydroxy-4-methoxybenzaldehyde (0.28 mmol); isolated as a yellow solid (35 mg, 36%); mp: 192 °C; ^1^H NMR (250 MHz, CDCl_3_) δ 9.72 (br s, 1H), 8.35 (s, 1H), 7.38 (d, *J* = 8.8 Hz, 1H), 7.35–7.22 (m, 3H), 7.20–7.11 (m, 2H), 6.67 (d, *J* = 2.6 Hz, 1H), 6.60 (dd, *J* = 8.8, 2.6 Hz, 1H), 4.00–3.89 (m, 2H), 3.85 (s, 3H), 3.83 (s, 3H), 2.93 (t, *J* = 7.3 Hz, 2H), 2.27 (s, 3H), ^13^C NMR (63 MHz, CDCl_3_) δ 168.1, 164.9, 164.3, 161.1, 148.9, 141.4, 138.0, 137.9, 129.0 (2C), 128.9 (2C), 127.01, 121.7, 118.8, 109.5, 105.4, 105.4, 55.7, 51.2, 42.7, 35.4, 12.8.; IR (neat, cm^−1^): 1651.1, 1588.5, 1286.1, 1195.3; elemental analysis (%) calculated for C_23_H_23_NO_5_: C, 70.21; H, 5.89; N, 3.56. Found: C, 69.92; H, 5.79; N, 3.42.

#### 2.4.9. Methyl (*Z*)-4-(3,4-Dihydroxybenzylidene)-2-methyl-5-oxo-1-phenethyl-4,5-dihydro-1*H*-pyrrole-3-carboxylate (**3i**) 

Prepared from pyrrolinone **1b** (0.25 mmol) and 3,4-dihydroxybenzaldehyde (0.28 mmol); isolated as a yellow solid (23 mg, 24%); mp: 159–160 °C; ^1^H NMR (250 MHz, CDCl_3_) δ 8.23 (d, *J* = 1.8 Hz, 1H), 8.15 (s, 1H), 7.45 (dd, *J* = 8.3, 1.7 Hz, 1H), 7.32–7.19 (m, 3H), 7.17–7.09 (m, 2H), 6.92 (d, *J* = 8.3 Hz, 1H), 3.98–3.75 (m, *J* = 9.3 Hz, 5H), 2.87 (t, *J* = 7.4 Hz, 2H), 2.24 (s, 3H); ^13^C NMR (63 MHz, CDCl_3_) δ 166.2, 165.1, 150.7, 147.5, 143.6, 142.9, 138.2, 129.0 (2C), 128.9 (2C), 127.9, 127.6, 127.0, 124.1, 118.4, 115.0, 104.3, 51.2, 42.3, 35.5, 13.1; IR (neat, cm^−1^): 1689.9, 1650.4, 1595.7, 1289.7, 1169.6; elemental analysis (%) calculated for C_22_H_21_NO_5_: C 69.65, H 5.58, N 3.69; found: C 69.31, H 5.31, N 3.49.

#### 2.4.10. Methyl (*Z*)-4-[4-((2-Fluorobenzyl)oxy)-3-methoxybenzylidene]-2-methyl-5-oxo-1-phenethyl-4,5-dihydro-1*H*-pyrrole-3-carboxylate (**3j**) 

Prepared from pyrrolinone **1b** (0.25 mmol) and aldehyde **2c** (0.28 mmol); isolated as a yellow solid (35 mg, 28%); mp: 94–95 °C; ^1^H NMR (250 MHz, CDCl_3_) δ 8.29 (d, *J* = 2.0 Hz, 1H), 8.14 (s, 1H), 7.63 (dd, *J* = 8.7, 1.8 Hz, 1H), 7.52 (td, *J* = 7.5, 1.7 Hz, 1H), 7.35–7.26 (m, 3H), 7.15 (m, 4H), 6.93 (d, *J* = 8.5 Hz, 1H), 6.86 (d, *J* = 11.2 Hz, 1H), 5.30 (s, 2H), 3.99 (s, 3H), 3.89–3.81 (m, 5H), 2.90 (t, *J* = 7.2 Hz, 2H), 2.21 (s, 3H); ^13^C NMR (63 MHz, CDCl_3_) δ 166.0, 165.1, 160.4 (d, *J* = 246.1 Hz), 151.5, 149.9, 148.8, 141.8, 138.4, 129.8 (d, *J* = 8.14 Hz),129.6 (d, *J* = 3.9 Hz), 129.1 (2C), 128.8 (2C), 128.7, 127.2, 126.9, 124.8, 124.5 (d, *J* = 3.6 Hz), 124.0 (d, *J* = 14.2 Hz), 115.4 (d, *J* = 21.1 Hz), 115.2, 112.5, 103.7, 64.4 (d, *J* = 4.6 Hz), 56.3, 51.0, 42.2, 35.5, 13.2; IR (neat, cm^−1^): 1675.5, 1586.8, 1267.7, 1140.2; elemental analysis (%) calculated for C_30_H_28_FNO_5_: C 71.84, H 5.63, N 2.79; found: C 71.64, H 5.62, N 2.81.

#### 2.4.11. Methyl (*Z*)-4-[4-((2-Chlorobenzyl)oxy)-3-methoxybenzylidene]-2-methyl-5-oxo-1-phenethyl-4,5-dihydro-1*H*-pyrrole-3-carboxylate (**3k**) 

Prepared from pyrrolinone **1b** (0.25 mmol) and aldehyde **2b** (0.28 mmol); isolated as a yellow solid (47 mg, 37%); mp: 112–113 °C; ^1^H NMR (250 MHz, CDCl_3_) δ 8.31 (d, *J* = 2.0 Hz, 1H), 8.14 (s, 1H), 7.65–7.51 (m, 2H), 7.43–7.36 (m, 1H), 7.33–7.13 (m, 7H), 6.86 (d, *J* = 8.5 Hz, 1H), 5.34 (s, 2H), 4.01 (s, 3H), 3.90–3.76 (m, 5H), 2.90 (t, *J* = 7.3 Hz, 2H), 2.21 (s, 3H); ^13^C NMR (63 MHz, CDCl_3_) δ 166.0, 165.1, 151.5, 149.9, 148.8, 141.8, 138.4, 134.5, 132.3, 129.5, 129.1, 129.1 (2C), 128.9 (2C), 128.7, 128.6, 127.2 (2C), 126.9, 124.8, 115.2, 112.6, 103.7, 67.8, 56.3, 51.0, 42.2, 35.5, 13.2; IR (neat, cm^−1^): 1674.3, 1586.3, 1274.1; elemental analysis (%) calculated for C_30_H_28_ClNO_5_: C 66.49, H 5.58, N 2.42; found: C 66.65, H 5.38, N 2.60.

#### 2.4.12. Methyl (*Z*)-4-[4-((4-Chlorobenzyl)oxy)-3-methoxybenzylidene]-2-methyl-5-oxo-1-phenethyl-4,5-dihydro-1*H*-pyrrole-3-carboxylate (**3l**) 

Prepared from pyrrolinone **1b** (0.25 mmol) and aldehyde **2a** (0.28 mmol); isolated as a yellow solid (49 mg, 38%); mp: 68–70 °C; ^1^H NMR (250 MHz, CDCl_3_) δ 8.21 (d, *J* = 2.0 Hz, 1H), 8.06 (s, 1H), 7.54 (dd, *J* = 8.7, 1.9 Hz, 1H), 7.37–7.05 (m, 9H), 6.78 (d, *J* = 8.5 Hz, 1H), 5.10 (s, 2H), 3.91 (s, 3H), 3.81–3.72 (m, 5H), 2.82 (t, *J* = 7.3 Hz, 2H), 2.13 (s, 3H); ^13^C NMR (63 MHz, CDCl_3_) δ 165.9, 165.1, 151.5, 149.9, 148.8, 141.7, 138.4, 135.3, 133.8, 129.1, 128.9, 128.8, 128.7, 127.0, 126.9, 124.8, 115.2, 112.6, 103.7, 70.0, 56.2, 51.0, 42.1, 35.5, 13.2; IR (neat, cm^−1^): 1686.2, 1592.8, 1260.3; elemental analysis (%) calculated for C_30_H_28_ClNO_5_: C 69.56, H 5.45, N 2.70; found: C 69.22, H 5.27, N 2.68.

#### 2.4.13. Methyl (*Z*)-4-[4-((4-Chlorobenzyl)oxy)-3-methoxybenzylidene]-1-(3,4-dimethoxyphenethyl)-2-methyl-5-oxo-4,5-dihydro-1*H*-pyrrole-3-carboxylate (**3m**)

Prepared from pyrrolinone **1c** (0.25 mmol) and aldehyde **2a** (0.28 mmol); isolated as a yellow solid (62 mg, 43%); mp: 70 °C; ^1^H NMR (250 MHz, CDCl_3_) δ 8.06 (d, *J* = 2.0 Hz, 1H), 7.94 (s, 1H), 7.41 (dd, *J* = 8.6, 1.8 Hz, 1H), 7.21–7.09 (m, 5H), 7.04 (s, 1H), 6.70–6.40 (m, 6H), 4.97 (s, 2H), 3.78 (s, 3H), 3.68–3.55 (m, 11H), 2.65 (t, *J* = 6.7 Hz, 2H), 1.98 (s, 3H); ^13^C NMR (63 MHz, CDCl_3_) δ 166.0, 165.0, 151.6, 149.9, 149.1, 148.8, 145.0, 141.7, 135.3, 133.9, 131.0, 128.9, 128.8, 128.7, 127.0, 124.9, 121.0, 115.2, 112.7, 112.2, 111.5, 103.6, 70.1, 56.2, 56.0, 56.0, 51.0, 42.3, 35.0, 13.3; IR (neat, cm^−1^): 1685.9, 1590.4, 1259.1, 1248.0; elemental analysis (%) calculated for C_32_H_32_ClNO_7_: C 66.49, H 5.58, N 2.42; found: C 66.74, H 5.41, N 2.41.

#### 2.4.14. Methyl (*Z*)-4-[4-((2-Chlorobenzyl)oxy)-3-methoxybenzylidene]-1-(3,4-dimethoxyphenethyl)-2-methyl-5-oxo-4,5-dihydro-1*H*-pyrrole-3-carboxylate (**3n**)

Prepared from pyrrolinone **1c** (0.25 mmol) and aldehyde **2b** (0.28 mmol); isolated as a yellow solid (67 mg, 46%); mp 100–101 °C ^1^H NMR (250 MHz, CDCl_3_) δ 8.23 (d, *J* = 2.0 Hz, 1H), 8.08 (s, 1H), 7.55 (dd, *J* = 8.7, 1.8 Hz, 1H), 7.51–7.43 (m, 1H), 7.35–7.29 (m, 1H), 7.22–7.15 (m, 2H), 6.79 (d, *J* = 8.5 Hz, 1H), 6.72 (d, *J* = 8.2 Hz, 1H), 6.64 (dd, *J* = 8.1, 1.9 Hz, 1H), 6.56 (d, *J* = 1.9 Hz, 1H), 5.26 (s, 2H), 3.94 (s, 3H), 3.81–3.71 (m, 11H), 2.78 (t, *J* = 7.1 Hz, 2H), 2.12 (s, 3H); ^13^C NMR (63 MHz, CDCl_3_) δ 166.0, 165.0, 151.5, 149.9, 149.1, 148.8, 147.9, 141.8, 134.5, 132.3, 131.0, 129.4, 129.0, 128.7, 128.6, 127.2 (2C), 124.8, 121.0, 115.2, 112.6, 112.2, 111.4, 103.6, 67.8, 56.3, 56.0, 56.0, 51.0, 42.3, 35.0, 13.3; IR (neat, cm^−1^): 1685.9, 1590.2, 1260.3, 1232.4; elemental analysis (%) calculated for C_32_H_32_ClNO_7_: C 66.49, H 5.58, N 2.42; found: C 66.65, H 5.38, N 2.60.

#### 2.4.15. Methyl (*Z*)-4-((1*H*-Indol-3-yl)methylene)-2-methyl-5-oxo-1-phenethyl-4,5- dihydro-1*H*-pyrrole-3-carboxylate (**3o**) 

Prepared from pyrrolinone **1b** (0.25 mmol) and indol-3-carboxaldehyde (0.28 mmol); isolated as a yellow solid (28 mg, 30%); mp: 223–224 °C; ^1^H NMR (250 MHz, *d_6_*-DMSO) δ 12.08 (br s, 1H), 9.42 (d, *J* = 2.8 Hz, 1H), 8.56 (s, 1H), 7.83–7.71 (m, 1H), 7.58–7.48 (m, 1H), 7.38–7.12 (m, 7H), 3.89 (t, *J* = 7.3 Hz, 2H), 3.81 (s, 3H), 2.87 (t, *J* = 7.3 Hz, 2H), 2.32 (s, 3H); ^13^C NMR (63 MHz, *d_6_*-DMSO) δ 165.3, 164.5, 148.8, 138.6, 136.0, 133.2, 129.5, 128.9 (2C), 128.5 (2C), 128.4, 126.5, 122.6, 121.0, 119.3, 117.4, 112.5, 111.7, 102.1, 50.8, 41.3, 34.7, 12.6; IR (neat, cm^−1^): 1702.4, 1583.3, 1326.7, 1230.4; elemental analysis (%) calculated for C_24_H_22_N_2_O_3_: C 68.64, H 6.22, N 3.20; found: C 68.49, H 6.02, N 3.32.

### 2.5. Antioxidant Assesment by the 1,1-Diphenyl-2-picryl-hydrazyl (DPPH) Method

The direct antioxidant effects of compounds **3** were investigated by studying their radical-scavenging effect on the 2,2-diphenyl-1-picrylhydrazyl free radical (DPPH^•^). Thus, a 100 μL volume of a methanol solution of each compound was added to 200 μL of 100 μM methanolic DPPH^•^, and the solution was kept in the dark at 37 °C for 30 min in a 96-well microliter plate. After this incubation, the absorption at 490 nm of each solution was measured with a microplate reader in order to quantitate its decrease. A blank reading was also taken to calculate the amount of remaining DPPH^•^. The radical-scavenging activity exerted by compounds **3** was determined by comparison with a 10 μL of MeOH-treated blank group. Ascorbic acid, a well-known antioxidant acting as a radical scavenger, was used as reference. All samples were analyzed in triplicate. Percent inhibition of the DPPH^•^ radical was calculated using the equation shown below, where Abs_Ctrl_ is the absorbance of the blank group and Abs_s_ is the absorbance of the samples containing compounds **3**. For each compound showing a percentage of inhibition above 60%, the IC_50_ value was determined using a dose–response curve obtained from measurements corresponding to at least six concentrations.

$$\%Scavenger=\frac{\left({Abs}_{Ctrl}-{Abs}_{S}\right)}{{Abs}_{Ctrl}}\times 100$$

### 2.6. Antioxidant Assesment by the Ferric Reducing Antioxidant Power (FRAP) Method

A ferric reducing antioxidant power (FRAP) kit assay purchased from Sigma-Aldrich (Madrid, Spain) was employed for the experiments. The antioxidant solutions, FRAP assay buffer solution, FeCl_3_ solution, and FRAP probe solution were kept in ice during the preparation of the plate. A calibration standard curve was prepared by using the 2 mM ferrous standard solution provided in the FRAP kit assay. Appropriate aliquots of this solution were taken and diluted up to a 200 μL final volume with FRAP buffer. The final ferrous concentrations measured in the well were: 0, 20, 40, 60, 80, and 100 μM. Each concentration value was prepared and measured by triplicate. Stock solutions of compounds **3**, as well as reference compounds trolox and ferulic acid, were freshly prepared in 95% ethanol at 30 μM concentration. Aliquots of 10 μL of compounds **3** and the reference compounds were mixed with 19 μL FeCl_3_ solution and 19 μL FRAP probe solution, and then completed to a final volume of 200 μL with FRAP assay buffer solution. The final concentration of antioxidants and references was 30 μM. Compounds **3**, reference compounds, as well as the corresponding blank solutions (one for each compound to be assayed, avoiding the chromogenic FRAP probe), and positive control were thoroughly mixed in vortex for 1–2 min with the reaction chromogenic media, and then were incubated under darkness at 37 °C in the dry bath, and using the device for support well-plates. Each compound to be assayed and the reference antioxidant compounds were prepared and measured by triplicate. The absorbance values were measured at 594 nm after 5 min of reaction and, to assure complete reaction, the absorbance values after 60 min of incubation at 37 °C in the dry bath were also measured. The absorbance values of the sample antioxidant compounds were corrected by subtracting the corresponding absorbance values of the blanks, and then they were introduced in the calibration curve and the ferrous equivalents were determined. The reference antioxidants employed for this assay were trolox and ferulic acid, which were obtained from Sigma-Aldrich (Madrid, Spain). Ultrapure water was obtained from a Milli-Q Direct 8 system (Millipore, Molsheim, France). 

### 2.7. Acetylcholinesterase (AChE) Inhibition Assay

Ellman’s method was employed to determine the inhibitory capacity of the compounds against AChE (*Electrophorus electricus*) [18]. The compounds were studied at 10 μM concentration, at room temperature, using PBS (0.1 M, pH 8) as solvent. Transparent 48-well microplates (COSTAR 3548) were used and, in each one, a blank was included. The compounds were incubated with the enzyme (0.09 U/mL) and with DTNB (0.35 mM) for 10 min. Afterwards, the enzyme substrate acetylthiocholine (0.35 mM) was added to a final volume of 1 mL. The microplate was incubated at room temperature for 15 min. Then, the absorbance was measured at 420 nM using the microplate reader FluoStar Optima (BMG Labtech, Ortenberg, Baden-Württemberg, Germany). The enzymatic activity was calculated by subtracting the blank absorbance from the sample absorbance and dividing the result by the absorbance of the maximum activity subtracted from blank absorbance, and the result was multiplied by one hundred to give a percentage. The inhibitory activity was calculated by subtracting the enzymatic activity from one hundred. Selected compounds were assayed at 0.3, 1, 3, 10, and 30 μM concentrations to obtain inhibition curves. The results were plotted versus concentration to obtain IC_50_ values through sigmoidal non-lineal adjustment. All the experiments were performed at least in triplicate.

### 2.8. Determination of Nrf2 Transcription Factor Induction

AREc32 cells (kindly shared by Dr. Professor Ronald C. Wolf, University of Dundee, Dundee, UK) were grown in Dulbecco’s modified Eagle medium (DMEM) with glutamax, supplemented with 10% fetal bovine serum (FBS), 1% of antibiotics penicillin–streptomycin, and geneticin (0.8 mg/mL, G418) (GIBCO, Madrid, Spain). Cells were harvested in a 75 cm^3^ flask with 11 mL of the specified medium, incubated at 37 °C and 5% CO_2_, and they were transferred to a new flask every 4–6 days, when the confluence was around 80%. Cells were seeded in 96-well white plates (20,000 cells/well) using 100 μL/well. After 24 h, cells were treated with compounds at desired concentrations (1, 5, 10, 15 μM) for 24 h, including *tert*-butyl hydroquinone (TBHQ, 10 μM) as a positive control. Another 24 h later, luciferase activity was measured through a bioluminescence assay using a “Luciferase assay system” (Promega E1500, Madison, WI, USA), following the protocol given by the provider, and quantifying luminescence in an Orion II microplate luminometer (Berthold, Germany). Measurements were performed by duplicate, and the values were normalized to basal luminescence considered as 1. Nrf2 induction potency was expressed as CD values, i.e., the concentrations required to double the luciferase activity, and are calculated from dose–response curves generated by plotting fold induction of control conditions vs. compound concentration, and fitted by non-linear regression and data interpolated to value 2.

### 2.9. Immunocytochemistry

AREc32 or SH-SY5Y cells were seeded in 24 multiwell plates (40,000 or 100,000 cells per well, respectively) on poly-D-Lysine coated crystal slides. AREc32 cells were incubated for 1 h with the compounds under assay, and were then fixed by treatment with a 4% solution of paraformaldehyde in PBS for 15 min, followed by three washings with PBS in 5 min intervals. SH-SY5Y cells were treated with compounds during 24 h, and then co-incubated with medium (basal), okadaic acid (OA, 20 nM), or OA plus compound **3i** (10 μM). Thereafter, cells were permeabilized by treatment with 0.5% Triton X-100 for 5 min, followed by three washings with PBS, and were incubated overnight with primary antibody anti-Nrf2 (ARE, 1:50, A-10, sc-365949, Santa Cruz Biotechnology, Dallas, TX, USA), anti-pTau AT8 (1:400, MN1020, Thermofisher, Madrid, Spain), and anti-HO-1 (1:400, Ab13243, Abcam, Madrid, Spain). After three washes with PBS, the cells were incubated with secondary antibodies (1:800, 1 h). In order to visualize the nuclei, the cells were counterstained during the second wash with 5 μg·mL^−1^ of Hoechst 33342 (Invitrogen, Madrid, Spain). Finally, the slides were protected with coverslips, glycerol-PBS (1:1 *v*/*v*) was added, and they were viewed with a confocal microscope (TCS SPE, Leica, Wetzlar, Germany).

### 2.10. Western Blot Analysis

AREc32 cells were collected from plates after 24 h of treatment with the selected compound at 30 µM. Then, they were lysed in ice-cold lysis buffer (1% Nonidet P-40, 10% glycerol, 137 mM NaCl, 20 mM Tris HCl pH 7.5, 1 μg/mL leupeptin, 1 mM phenylmethylsulfonyl fluoride, 20 mM NaF, 1 mM sodium pyrophosphate, and 1 mM Na_3_VO_4_). After this treatment, proteins (30 μg) were resolved by gel electrophoresis on sodium dodecyl sulfate–polyacrylamide (12%) and transferred to Immobilon-P membranes (MilliporeSigma, Burlington, MA, USA). The membranes were incubated with anti-GCLc (1:1000, Ab41463 (Abcam, Cambridge, MA, USA), anti−HMOX1 (1:1000, ab68477, Abcam), anti-NQO1 (1:1000, 11451-1-AP, Proteintech, Manchester, UK), or anti-β-Actin (1:100,000, A3854, Merck, Madrid, Spain). Peroxidase-conjugated secondary antibodies (1:10,000 and anti-rabbit: SC-2357, Santa Cruz Biotechnology, Dallas, TX, USA) were employed to detect the proteins by enhanced chemoluminescence. Band intensities corresponding to immunoblot detection of protein samples were quantitated with Fiji software [19].

### 2.11. Culture of SH-SY5Y Cells

SH-SY5Y cells (ECACC 94030304, Porton Down, Salisbury, UK), from passages between 4 and 16 after defreezing, were maintained in a MEM/F12 containing 15 nonessential amino acids and supplemented with 10% fetal bovine serum (FBS), glutamine 1 mM, 50 units/mL of penicillin, and 50 μg/mL of streptomycin (GIBCO, Madrid, Spain). Cells were harvested in a 75 cm^3^ flask and incubated at 37 °C with wet atmosphere and 5% CO_2_, carrying out passages 1:4 twice a week. For neuroprotection experiments, cells were seeded in 96-well plates at a density of 60,000 cells/well. For the toxicity experiments, cells were treated with the compounds under assay before reaching confluence, in MEM/F12 with 1% FBS. 

### 2.12. SH-SY5Y In Vitro Neuroprotection Studies

Neuroprotection studies against tau hyperphosphorylation exerted by okadaic acid (OA) were evaluated using the SH-SY5Y cell line as follows. Cells were preincubated with the compounds under assay at 1 μM for 24 h. After that time, the medium was removed and substituted by medium at 1% with FBS, the compounds at a concentration of 1 µM, and OA at 20 nM. After 18 h, cell viability was addressed by the MTT reduction method. MTT (5 mg/mL, 10 μL/well) was added and incubated for 2 h. Then, the medium was removed and the purple formazan crystals formed by viable cells were dissolved using 100 μL of DMSO. A FluoStar Optima spectrophotometer (BMG Labtech, Ortenberg, Baden-Württemberg, Germany) was used to measure the absorbance at 570 nm. Melatonin (1 µM) was used as a positive control and for comparative purposes. 

To evaluate the compounds for their neuroprotective effect in an oxidative stress model, we selected the rotenone (30 μM) and oligomycin A (10 μM) (R/O) cocktail, which disturbs the flow of electrons in the mitochondrial respiratory chain. Similarly, SH-SY5Y cells were preincubated with compounds **3** at 1 μM for 24 h. Then, they were co-incubated in the presence of R/O for a further 24 h, evaluating cell viability by the MTT reduction method. Melatonin (1 μM) was used as a positive control.

### 2.13. Reactive Oxygen Species Production

ROS production was evaluated using the fluorescent dye H_2_DCFDA [20]. Briefly, after deacetylation inside the cell, the H_2_DCF derivative is able to react with oxygen-free radicals and is transformed into dichlorofluorescein, increasing its fluorescence intensity. SH-SH5Y cells were seeded in 96-well clear-bottom black plates 6 × 10^4^ cells/well. After 24 h, cells were treated with compound **3i** at the desired concentrations for 24 h. Treatments were then eliminated and the cells were loaded with H_2_DCFDA (10 μM) for 45 min. Then, they were washed twice with Krebs–HEPES solution and were finally treated with the R/O (30/10 μM, respectively) toxic combination for 6 h in the presence of DMSO (toxic), or with increasing concentrations of compound **3i** (0.1, 1, and 10 μM). Fluorescence was evaluated in a fluorescence microplate reader (Fluostar optima; BMG Labtech, Ortenberg, Baden-Württemberg, Germany) at 485/520 excitation/emission wavelengths, respectively. Data are expressed as the increase after 5 h exposure to ROS generator, subtracting basal fluorescence at *t* = 0 h, and normalized to basal conditions as 100% production.

### 2.14. Superoxide Production Measurement

To evaluate superoxide production, we used the fluorescent dye dihydroethidium (DHE). SH-SY5Y cells were seeded in 96-well clear-bottom black plates (6 × 10^4^ cells/well). After 24 h, cells were treated with compound **3i** at the desired concentrations for 24 h. Thereafter, treatments were eliminated and cells were loaded with DHE (20 μM) for 45 min. Then, cells were washed twice with Krebs–HEPES solution and finally treated with the R/O (30/10 μM, respectively) toxic combination for 5 h in the presence of DMSO (toxic), melatonin (1 μM, positive control), or increasing concentrations of compound **3i** (1 and 10 μM). Fluorescence was evaluated in a Nikon T1000 fluorescence microscope at 490/630 excitation/emission wavelengths, respectively. Images were quantitated using Fiji software, and data are expressed as the increase after 6 h exposure to ROS generator, normalized to basal conditions as 100% increase.

### 2.15. Statistical Analysis

All numerical values are given as mean ± S.E.M. The IC_50_ and LD_50_ parameters were calculated from individual concentration–response curves by performing non-linear regression analysis using GraphPad Prism software (San Diego, CA, USA). The results were analyzed by comparing experimental and control data using one-way ANOVA, followed by Newman–Keuls post hoc test when three groups are implicated. Differences were considered to be statistically significant if *p* ≤ 0.05; “*n*” represents the number of different cultures used or enzyme inhibition assays performed.

## 3. Results

### 3.1. Synthesis of Bisavenantramide Analogs ***3***

**Figure 3:**
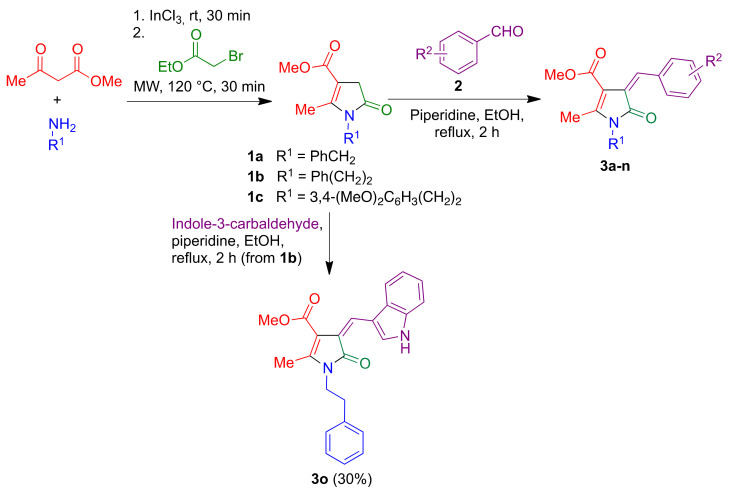
Synthesis of compounds **3**.

Pyrrolin-5-ones **1** were obtained via a sequential three-component process previously developed by our group [16], and were treated with the suitable aromatic aldehydes **2** via a Knoevenagel condensation in the presence of piperidine to afford arylmethylenepyrrolones **3** (Figure 3 and Table 1). The noncommercially available aldehydes, bearing 3-benzyloxy substituents and serving as precursors to compounds **3j**–**n**, were synthesized using a Williamson reaction. The choice of these particular side chains was inspired by their presence in neuroprotectant molecules with tau protein aggregation inhibitory properties [21].

### 3.2. Computational Druggability Study of Compounds ***3***

Preliminary computational studies of compounds **3** were carried out using the web tool SwissADME [22]. Generally speaking, the compounds showed good drug-like properties, with TPSA (topological polar surface area) values below 90 Å^2^. Most compounds satisfy Lipinski’s rule, with the exception of those bearing a substituted 4-benzyloxy group at R^2^ (compounds **3j**–**3n**), which have an MW above 500 and lipophilicity above 5 (Table S1 in Supplementary Materials). A PAINS alert appears due to the presence of a carbonyl-conjugated exocyclic double bond, but this feature is needed for analogy with the natural model and for Nrf2 induction. As shown in Figures S1 and S2, a good oral bioavailability is predicted for all compounds, and most of them are also predicted to cross the blood–brain barrier, with **3f** and **3i** being borderline. Compounds **3j**–**3n**, due to their high lipophilicity, are predicted to be substrates of the P-gp-170 transport protein, but the other compounds are exempt from this problem.

### 3.3. Characterization of Bisavenantramide Analogs ***3*** as Antioxidants

The radical scavenging activity of all compounds was first studied by the DPPH assay, which measures the ability of antioxidant compounds to donate a hydrogen atom to the violet 2,2-diphenyl-1-picrylhydrazyl radical and transform it into a nonradical species. Thus, it is generally considered to belong to the hydrogen atom transfer (HAT) category of methods for measurement of antioxidant activity [23]. Compounds **3a**–**o** were initially assessed at two concentrations (0.1 and 1 mM), and IC_50_ values were calculated for the more active compounds (Table 2). Two well-known anti-oxidant compounds, namely melatonin and ascorbic acid, were employed as references, and trolox was used as a positive control. In this assay, only phenolic compounds **3h** and **3i** showed a high radical scavenging activity, similar to that of ascorbic acid, while melatonin was inactive. 

In order to achieve a broader insight into the antioxidant profile of our compounds, we also examined them using a single electron transfer (SET)-based method, namely the ferric reducing antioxidant power (FRAP) assay, which measures the ability of an antioxidant to reduce the ferric ion in the TPTZ (2,4,6-tri(2-pyridyl)-1,3,5-triazine)-Fe(III) complex. The results obtained were expressed using trolox as a reference and ferulic acid as a positive control (Table 2), and show comparable activities (0.6 to 1.1 trolox units) for all compounds. Not surprisingly, the highest antioxidant activity was again found in the catechol derivative **3i**, which was more active than ferulic acid and slightly more active than trolox. 

### 3.4. Acetylcholinesterase (AChE) Inhibitory Activity

Bisavenanthramide B-6 and heliotropamide A have been identified as acetylcholinesterase inhibitors [8]. This precedent, coupled with our interest in the potential characterization of our compounds as multitarget-directed ligands, led us to investigate them as inhibitors of this enzyme, an important anti-Alzheimer target. The full library was first studied at 10 μM concentration, and IC_50_ values were also measured for the most promising compounds (Table 3). Only three of them showed activity in this assay, although moderate, and for this reason we did not further pursue this aspect of the pharmacological characterization of compounds **3**.

### 3.5. Biological Evaluation of Bisavenantramide Analogues

#### 3.5.1. Cytotoxicity Evaluation in the SHSY5Y Cell Line 

Due to their presumed electrophilicity, the neurotoxicity of our compounds was investigated by investigating their influence on cell viability in the neuroblastoma SHSY5Y cell line prior to other studies in cell cultures. Toxicity was measured as MTT reduction, and most compounds showed a good safety profile with an LD_50_ above 100 µM. Compound **3i**, showing LD_50_ = 92.8 ± 4.6 µM, can also be considered to have a suitable toxicity profile, but **3a** and **3c** were regarded as too toxic (Table 4). It is relevant to note that, despite their reputation for toxicity, interest in covalent drugs has undergone a renaissance, and about 30% of the recently marketed drugs belong to this class [24,25].

#### 3.5.2. Nrf2 Induction

The ability of our compounds to induce Nrf2 was evaluated in the AREc32 cell line, an MCF7 stable clone that is transfected with eight copies of the luciferase reporter gene immediately after the ARE sequence [26]. Therefore, Nrf2 activation and the subsequent transcription of the genes dependent on the ARE element also leads to expression of luciferase, which can be quantitated using a luminescence assay. AREc32 cells were incubated for 24 h with 0.3, 3, 10, 30, and 60 μM concentrations of compounds **3**, and a luminescence measurement allowed CD values to be obtained. This parameter characterizes the Nrf2-inducing ability of each compound and represents the concentration required to double the luciferase activity compared to its basal expression. The results collected in Table 5 prove that all compounds are good Nrf2 inducers, and some of them, namely **3g**, **3i**, and **3k**, displayed excellent activities below 10 μM in CD value. Compound **3g** was the most potent one, with a CD value of 3.18, which represents a higher potency than the potent natural Nrf2 inductor caffeic acid, whose literature CD value is comprised between 6.5 and 11.75 μM [27].

#### 3.5.3. Compound **3g** Induces Nrf2 Nuclear Translocation and Nrf2-ARE Dependant Protein Expression

**Figure 4:**
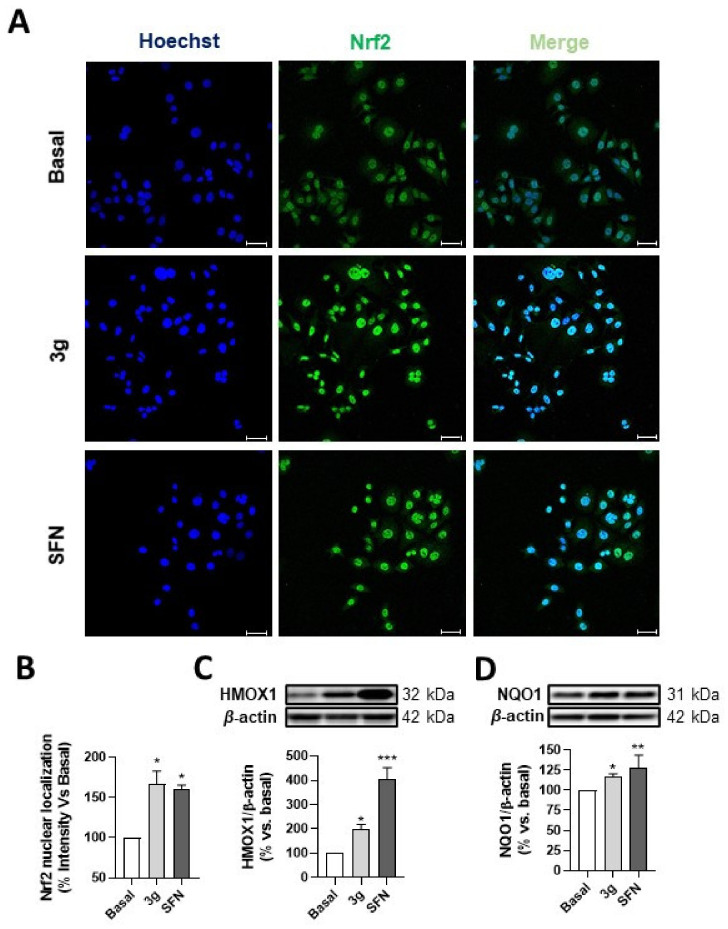
Compound **3g** effectively induces Nrf2 translocation and overexpression of Nrf2-ARE dependent genes in AREc32 cells. (**A**) AREc32 cells were treated with compound **3g** (30 µM), sulforaphane (10 µM), or culture medium (Basal) for 1 h; the cells were next processed for immunocytochemistry and stained with anti-Nrf2 (green) and Hoechst (blue). (**B**) Nrf2 nuclear localization analysis, measured as integrated intensity localized at the nucleus. HMOX1 (**C**) and NQO1 (**D**) expression in AREc32, measured by Western blot analysis. Cells were treated with compound **3g** (30 µM), sulforaphane (10 µM), or culture medium (basal) for 24 h and protein expression was then assessed. Graphs are expressed as densitometric quantification using β-actin for normalization (bottom). Scale bar: 25 µm. Data are expressed as mean ± S.E.M. of six independent experiments. Statistical analysis was performed using one-way ANOVA: (*p* < 0.05); * *p* < 0.05; ** *p* < 0.01; *** *p* < 0.001 vs. basal condition.

After confirming the Nrf2 induction capability of compounds **3**, we were interested in carrying out an in-depth characterization of Nrf2 activation by compound **3g**, which had demonstrated the most potent activity to activate the phase II antioxidant response. To this end, we first studied Nrf2 nuclear translocation in the presence of compound **3g**. AREc32 cells were treated with compound **3g** (30 μM) or culture media (basal conditions), and we also included sulforaphane (SFN, 10 μM), a potent natural Nrf2 inducer, as a positive control. Thereafter, the cells were fixed and doubly stained with anti-Nrf2 and Hoechst 33342. As shown in Figure 4A, under basal conditions Nrf2 was predominantly distributed in the cytosol; however, cells treated with compound **3g** present a predominately nuclear distribution of Nrf2, similar to that observed for sulforaphane. As previously discussed, the structural features of compounds **3** led us to propose their potential Nrf2 induction capacity due to the presence of a Michael acceptor that would confer electrophilic properties to our compounds. It is well known that electrophilic compounds are able to covalently interact with several Cys residues present at Keap1, the Nrf2 negative regulator. This interaction induces a conformational change at Keap1, liberating Nrf2 and allowing it to translocate into the nucleus to initiate the ARE-dependent gene expression. Thus, these results indicate that compound **3g** could be acting in a similar fashion to other known Nrf2 inducers.

To demonstrate the phase II response activation by compound **3g**, we investigated its effect on NRF2-dependent protein expression. AREc32 cells were treated with compound **3g** (30 μM), culture media (basal conditions), or sulforaphane (SFN, 10 μM) as a positive control, for 24 h. Thereafter, cells were recovered and processed for protein expression analysis. The Western blot results showed that treatment with compound **3g** for 24 h significantly increased protein levels of HMOX1 and NQO1, in AREc32 cells. By showing elevated Nrf2 downstream protein levels, these data confirm the upregulation of the Nrf2 pathway. 

#### 3.5.4. Neuroprotection in a Rotenone/Oligomycin A Oxidative Stress Model

The fact that most of compounds **3** showed good Nrf2 induction, coupled with the radical scavenging properties found for some of them (**3h** and **3i**), prompted us to study the potential neuroprotective activity of our library in an oxidative stress model, which was generated by treatment of neuroblastoma SH-SY5Y cells with a combination of rotenone and oligomycin A (R/O) (30/10 μM). These compounds inhibit, respectively, complexes I and V of the electron transport chain at mitochondria, thereby disrupting ATP synthesis. Thus, the R/O combination constitutes a good model of oxidative stress and neurodegeneration with a mitochondrial origin, and has been broadly employed to evaluate potential hit compounds for the treatment of neurodegenerative diseases [28,29]. Furthermore, treatment of laboratory animals with rotenone is known to induce many features associated to Parkinson’s disease, such as selective nigrostriatal dopaminergic degeneration and the appearance of α-synuclein cytoplasmic inclusions, which are associated with oxidative damage [30,31,32]. 

**Figure 5:**
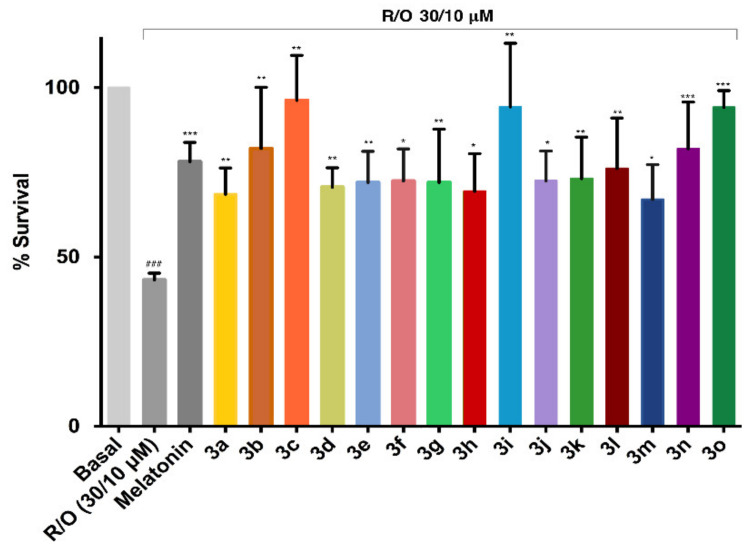
Survival (%) of SH-SY5Y cells subsequent to the administration of the combination of rotenone (30 µM) and olygomicin A (10 µM) in the presence of compounds **3a**–**3o** (1 µM), with melatonin as a positive control. Data were obtained from three experiments by triplicate and are given as mean ± SEM. One-way ANOVA Newman–Keuls post-test: ### *p* < 0.001 compared to basal; * *p* < 0.05, ** *p* < 0.01, *** *p* < 0.001 compared to toxic. Numerical data are given in the Supplementary Materials (Table S1).

Compounds **3a**–**o** were neuroprotective in a concentration-dependent manner against the rotenone–oligomycin oxidative insult (Figure 5 and Table S2). Many of the compounds (**3b**, **3c**, **3g**, **3i**, **3k**, **3l**, **3n**, **3o**) showed a level of neuroprotection similar to or higher than melatonin, a well-known antioxidant and neuroprotector [33]. Compounds **3c, 3i**, and **3o**, in particular, had an excellent neuroprotective profile, with >90% protection at 1 μM. 

#### 3.5.5. Neuroprotection against Tau Hyperphosphorylation Induced by Okadaic Acid

Okadaic acid is a potent inhibitor of protein phosphatase 2A (PP2A), thereby inducing tau hyperphosphorylation. For this reason, it can be employed as a model of neurodegenerative-disease-connected tau hyperphosphorylation, a well-known feature of Alzheimer’s disease, but present also in other NDDs, collectively known as tauopathies and characterized by the pathological aggregation of hyperphosphorylated tau in neurofibrillary tangles. A growing body of evidence shows the participation of tau pathology in many features of Parkinson’s disease, such as the aberrant hyperphosphorylation of tau protein, the presence in about 50% of PD patient brains of tau aggregates, which can be transported between neurons, and the tau/α-synuclein interaction [32]. Tau hyperphosphorylation is connected to oxidative stress, which activates p38 and deactivates calcineurin, a phosphatase involved in tau dephosphorylation by increasing RCAN1, its natural repressor [34,35]. Indeed, hyperphosphorylation and oxidative stress have been proposed to generate a vicious circle that is critical to the advance of neurodegenerative tauopathies [36].

**Figure 6:**
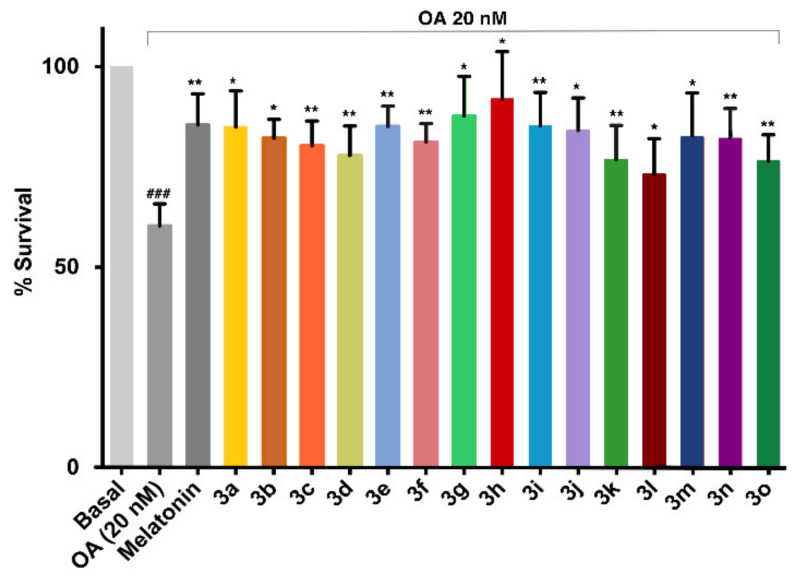
Survival (%) of SH-SY5Y cells following a toxic insult induced by okadaic acid (OA) (20 nM) in the presence of compounds **3a**–**3o** (1 µM), with melatonin as a positive control. Data are expressed as mean ± SEM of three experiments by triplicate. One-way ANOVA Newman–Keuls post-test: ### *p* < 0.001; compared to basal; * *p* < 0.05, ** *p* < 0.01, compared to the toxic. Numerical data are given in the Supplementary Materials (Table S2).

When compounds **3a**–**o** were administered to SH-SY5Y cells previously treated with okadaic acid, some of them (**3a**, **3e**, **3g**, **3h**) afforded a level of neuroprotection similar to melatonin. The best results corresponded to the phenolic compound **3h**, which provided an excellent neuroprotection against the okadaic-acid-induced toxicity (Figure 6 and Table S3).

#### 3.5.6. Compound **3i** Affords Neuroprotection by Reducing Free Radical Production and Tau Hyperphosphorylation and also by Nrf2 Induction

**Figure 7:**
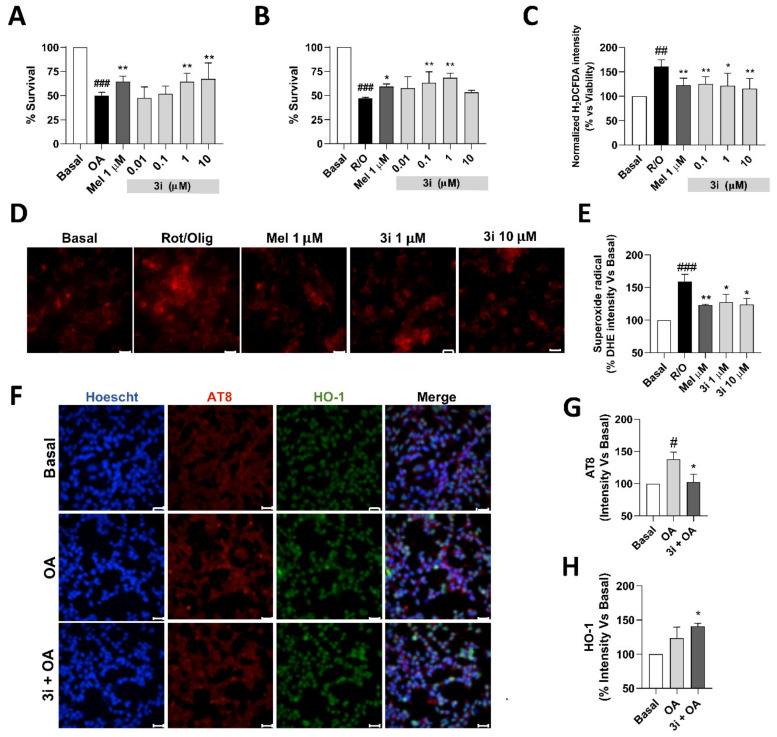
Neuroprotection capacity and mechanism of action of compound **3i**. (**A**) Survival (%) of SH-SY5Y cells following toxicity elicited by okadaic acid (20 nM) in the presence of increasing concentrations of **3i**. (**B**) Survival (%) of SH-SY5Y cells following toxicity elicited by okadaic acid (20 nM) in the presence of increasing concentrations of **3i**. (**C**) Total free radical production induced by toxic R/O combination for 6 h, measured as H_2_DCFDA fluorescence increase compared to basal conditions (DMSO). (**D**) Superoxide production induced by toxic R/O combination for 6 h, measured as DHE fluorescence increase, representative microphotographs (scale bar: 25 µm). (**E**) Quantification of DHE fluorescence increase compared to basal conditions. (**F**) Representative microphotographs (scale bar: 25 µm) of SH-SY5Y cells treated with **3i** (10 µM) during 24 h and then co-incubated with compound **3i** and OA (20 nM). Finally, cells were stained with Nuclei (Hoechst, Blue), phospho-tau (AT8, Red), and HO-1 (anti-HO-1, Green) and merge images. (**G**) Immunofluorescence quantification of phospho-tau production normalized to basal conditions considered as 100%. (**H**) Immunofluorescence quantification of HO-1 expression normalized to basal conditions considered as 100%. Data are expressed as mean ± SEM of 3 experiments in duplicate. One-way ANOVA Newman–Keuls post-test # *p* < 0.05; ## *p* < 0.01; ### *p* < 0.001; compared to basal. * *p* < 0.05, ** *p* < 0.01; compared to toxic stimuli.

Considering the promising overall pharmacological profile of compound **3i**, we selected this derivative for further evaluation. Firstly, we studied its potential concentration-dependent neuroprotective profile against tau-hyperphosphorylation. To this end, we tested compound **3i** at increasing concentrations (0.01, 0.1, 1, 10, and 30 µM) against the toxicity induced by okadaic acid (OA, 20 nM) in SH-SY5Y cells. As depicted in Figure 7A, compound **3i** exerted a concentration-dependent neuroprotective effect being statistically significant at 1 and 10 µM; however, neuroprotection was lost at 30 µM (data not shown). Similarly, we also performed the same experiment against the toxicity induced by increased oxidative stress in the presence of the rotenone/oligomycin A combination (30/10 µM, respectively). Interestingly, in this case, compound **3i** showed a statistically significant neuroprotective effect at 0.1 and 1 µM, but this activity was lost at 10 µM. The lack of a full concentration dependency of neuroprotection against different toxic stimuli is a common observation in the evaluation of multitarget compounds and has been related to the different potency of the molecules towards their various targets.

To further evaluate its mechanism of action, and considering the importance of exacerbated oxidative stress in neurodegenerative diseases, we evaluated its capacity to reduce free radical species production using the above-mentioned R/O toxic combination to generate ROS and measure the influence of **3i** in the total ROS production by using the H2DCFDA dye, and also the specific superoxide production by using the selective dye dihydroetidium (DHE). To evaluate total ROS production, SH-SY5Y cells were treated with compound **3i** (0,1, 1, and 10 µM) or melatonin (1 µM) for 24 h. Thereafter, cells were loaded with H2DCFDA for 45 min and treated with the R/O combination alone or co-incubated with compound **3i** or melatonin for 6 h. Interestingly, compound **3i** was able to reduce ROS production almost to basal levels at all concentrations tested, showing a similar capacity to melatonin. ROS production reduction by compound **3i** can be partially related to its neuroprotective character at 0.1 and 1 µM concentration, but it also shows the same reduction in ROS production at the highest concentration, where, as demonstrated in Figure 7B, the compound loses its neuroprotective capacity. Intrigued by these results, we also measured superoxide production in the same conditions. After 24 h, following pretreatment of cells with compound **3i** (1 and 10 µM) and melatonin, they were loaded with DHE for 45 min and then treated with the R/O toxic combination alone or in combination with **3i**. This experiment showed that compound **3i** was able to reduce superoxide production at both concentrations, with potency similar to melatonin. These results confirm that our compound is able to reduce total ROS and superoxide production induced by mitochondrial intoxication. This reduction might be related to a combination of direct scavenger effect and an increased expression of Nrf2-dependent antioxidant genes, such as HO-1.

Finally, we evaluated the capacity of compound **3i** to reduce tau hyperphosphorylation in the SH-SY5Y cellular line induced by okadaic acid (OA). Previously, we had demonstrated the capacity of this compound to reduce cellular death induced by OA, achieving the highest level of neuroprotection at 10 µM, and thus we selected this concentration. Cells were treated with **3i** (10 µM) during 24 h to induce the expression of Nrf2-dependant genes. Thereafter, cells were treated with medium (basal), OA (20 nM) or the OA–**3i** combination (20 nM and 10 µM, respectively) for 8 h. In this experiment, we were interested in assessing the ability of compound **3i** to reduce phosphorylated Tau without achieving high toxicity and to increase HO-1 as a reference of Nrf2 induction. As shown in Figure 7F, OA treatment induced a significant increase of AT8 (Ser202, Thr205 phospho-Tau), increasing its levels by 40% compared to basal conditions. Interestingly, compound **3i** significantly reduced phopho-tau levels to basal levels. Moreover, compound **3i** also increased HO-1 levels (Figure 7F,H) 46% compared to basal levels and also, to a higher extent, compared to OA treated cells, although this difference was not significant. These results indicate that the neuroprotective capacity of compound **3i** is connected to a reduced production of phopho-tau protein induced by OA toxicity. This capacity might be related to the induction of Nrf2-related genes, as demonstrated by HO-1 increased expression induced by compound **3i** in this neurotoxic model.

## 4. Conclusions

In summary, we have shown the interesting neuroprotective profile of a library of 4-(arylmethylene)-2-pyrrolin-5-one derivatives designed as simplified analogues of the bis-avenanthramides, a group of natural antioxidants having common structural features with caffeic and ferulic acid. Most of these compounds lack cytotoxicity and are good inductors of the Nrf2-ARE antioxidant response, as proved by the luciferase assay, by Nrf2 nuclear localization analysis, and by showing an increased expression of ARE-expressed proteins, such as HMOX1 and NQO1. Some compounds have good neuroprotective properties in situations related to oxidative stress, including treatment with the rotenone/oligomycin combination, and also against hyperphosphorylation induced by okadaic acid. Further characterization of the antioxidant properties of our compounds was performed on compound **3i** and, together with Nrf2 induction, it was shown to involve reduction of phosphorylated tau levels. This compound displayed a well-balanced multitarget profile and can be considered an interesting starting point for future hit-to-lead efforts towards the discovery of new candidates for the treatment of neurodegenerative diseases. 

## Figures and Tables

**Table 1:** Structures and yields of compounds **3a**–**n**.

Entry	Comp.	R^1^	R^2^	Yield, %
1	**3a**	C_6_H_5_CH_2_	H	50
2	**3b**	C_6_H_5_CH_2_	4-OMe	60
3	**3c**	C_6_H_5_CH_2_	4-Cl	45
4	**3d**	C_6_H_5_CH_2_CH_2_	3,4-OCH_2_O	60
5	**3e**	C_6_H_5_CH_2_CH_2_	2,4-(MeO)_2_	41
6	**3f**	C_6_H_5_CH_2_CH_2_	2,4,5-(MeO)_3_	47
7	**3g**	C_6_H_5_CH_2_CH_2_	3,4,5-(MeO)_3_	53
8	**3h**	C_6_H_5_CH_2_CH_2_	3-OH, 4-OMe	36
9	**3i**	C_6_H_5_CH_2_CH_2_	3,4-(OH)_2_	24
10	**3j**	C_6_H_5_CH_2_CH_2_	3-OMe, 4-(2-FC_6_H_4_CH_2_O)	28
11	**3k**	C_6_H_5_CH_2_CH_2_	3-OMe, 4-(2-ClC_6_H_4_CH_2_O)	37
12	**3l**	C_6_H_5_CH_2_CH_2_	3-OMe, 4-(4-ClC_6_H_4_CH_2_O)	38
13	**3m**	3,4(MeO)_2_C_6_H_3_CH_2_CH_2_	3-OMe, 4-(4-ClC_6_H_4_CH_2_O)	43
14	**3n**	3,4(MeO)_2_C_6_H_3_CH_2_CH_2_	3-OMe, 4-(2-ClC_6_H_4_CH_2_O)	46

**Table 2:** Radical scavenging properties of compounds **3** in the DPPH and FRAP assays.

Entry	Compound	DPPH			FRAP	
		Scavenging at 0.1 mM, %	Scavenging at 1 mM, %	IC_50_, μM	nmol Fe^2+^ at 30 µM	TEAC
1	**Trolox**			11.4 ± 1.0 (9)	2.28 ± 0.15 (9)	1.00
2	**Ascorbic acid**			16.2 ± 0.7 (9)		
3	**Melatonin**			1988 ± 1397 (2)		
4	**Ferulic acid**				1.98 ± 0.04 (3)	0.87
5	**3a**	9.7 ± 3.8	47.5 ± 4.9	-	1.41 ± 0. 03 (3)	0.62
6	**3b**	6.5 ± 4.5	27.2 ± 6.4	-	1.44 ± 0.02 (3)	0.63
7	**3c**	3.7 ± 3.8	36.6 ± 3.4	-	1.41 ± 0.03 (3)	0.62
8	**3d**	40.5 ± 5.0	41.6 ± 4.1	-	1.41 ± 0.04 (3)	0.62
9	**3e**	6.33 ± 4.2	27.0 ± 2.6	-	1.44 ± 0.01 (3)	0.63
10	**3f**	9.7 ± 0.6	9.7 ± 2.2	-	1.42 ± 0.04 (3)	0.62
11	**3g**	8.1 ± 4.7	46.5 ± 2.3	-	1.36 ± 0.10 (3)	0.60
12	**3h**	89.0 ± 0.4	-	26.3 ± 2.1 (3)	1.63 ± 0.04 (3)	0.71
13	**3i**	90.9 ± 0.5	-	7.5 ± 0.4 (3)	2.30 ± 0.08 (3)	1.01
14	**3j**	4.3 ± 2.1	31.3 ± 3.3	-	1.60 ± 0.04 (3)	0.70
15	**3k**	7.3 ±1.4	27.3 ± 1.0	-	1.63 ± 0.01 (3)	0.71
16	**3l**	11.3 ± 2.6	63.5 ± 2.2	712.2 ± 5.4 (3)	1.62 ± 0.03 (3)	0.71
17	**3m**	22.8 ± 0.2	85.5 ± 2.7	334.3 ± 22.9 (3)	1.59 ± 0.05 (3)	0.70
18	**3n**	12.7 ± 3.5	73.6 ± 0.7	552.1 ± 17.4 (3)	1.59 ± 0.02 (3)	0.69
19	**3o**	12.7 ± 1.9	49.9 ± 3.4	-	1.80 ± 0.07 (3)	0.79

**Table 3:** Anticholinesterase activity of compounds **3**.

Entry	Compound	% Inhibition of EeAChE at 10 μM	IC_50_ (μM)
1	**3a**	27.36	
2	**3b**	36.71	29.7 ± 3.0 (3)
3	**3c**	33.47	
4	**3d**	25.21	
5	**3e**	24.32	
6	**3f**	29.29	
7	**3g**	11.62	
8	**3h**	24.25	
9	**3i**	27.21	
10	**3j**	15.96	
11	**3k**	40.09	22.4 ± 3.2 (3)
12	**3l**	26.20	
13	**3m**	31.77	
14	**3n**	17.59	
15	**3o**	39.84	12.5 ± 1.4 (3)

**Table 4:** Toxicity of compounds **3** in neuroblastoma SHSY5Y cells.

Entry	Compound	LD_50_ (μM)
1	**3a**	63.7 ± 12.8
2	**3b**	>100
3	**3c**	75.4 ± 1 5.6
4	**3d**	>100
5	**3e**	>100
6	**3f**	>100
7	**3g**	>100
8	**3h**	>100
9	**3i**	92.8 ± 4.6
10	**3j**	>100
11	**3k**	>100
12	**3l**	>100
13	**3m**	>100
14	**3n**	>100
15	**3o**	>100

**Table 5:** Nrf2 induction by compounds **3**, as measured by the luciferase assay in the AREc32 cell line. CD is the compound concentration that doubles luciferase activity in comparison to its basal expression.

Entry	Compound	CD (μM)
1	**Caffeic acid**	6.5 to 11.75
2	**3a**	30.22 ± 1.89
3	**3b**	17.96 ± 1.84
4	**3c**	15.00 ± 0.80
5	**3d**	17.86 ± 8.58
6	**3e**	10.38 ± 0.80
7	**3f**	31.06 ± 6.21
8	**3g**	3.18 ± 1.16
9	**3h**	36.07 ± 418
10	**3i**	7.51 ± 3.45
11	**3j**	11.91 ± 2.83
12	**3k**	7.58 ± 4.35
13	**3l**	13.97 ± 4.45
14	**3m**	21.41 ± 5.72
15	**3n**	14.26 ± 3.89
16	**3o**	35.98 ± 10.56

## Data Availability

Data is contained within the article or Supplementary Materials.

## Supplementary Materials

The following are available online at https://www.mdpi.com/article/10.3390/antiox10060941/s1, Tables S1–S3, Figures S1 and S2 and copies of spectra of all new compounds.
