# Supplementary material for: Bisavenathramide Analogues as Nrf2 Inductors and Neuroprotectors in In Vitro Models of Oxidative Stress and Hyperphosphorylation
Source: Antioxidants (Basel). 2021 Jun 10;10(6):941. doi: 10.3390/antiox10060941 (PMC8230565; doi:10.3390/antiox10060941)

Supporting Information

Bisavenathramide analogues as Nrf2 inductors and neuroprotectors in *in vitro* models of oxidative stress and hyperphosphorylation

Ángel Cores [1], Sheila Abril [2], Patrycja Michalska [2], Pablo Duarte[2,3], Ana I. Olives, M. Antonia Martín, Mercedes Villacampa[1], Rafael León [2,3,*] and J. Carlos Menéndez [1,*]

1 Unidad de Química Orgánica y Farmacéutica, Departamento de Química en Ciencias Farmacéuticas. Facultad de Farmacia, Universidad Complutense, 28040 Madrid, Spain; acores@ucm.es (Á.C.), mvsanz@ucm.es (M.V.)
2 Instituto Teófilo Hernando y Departamento de Farmacología y Terapéutica, Facultad de Medicina, Universidad Autónoma de Madrid, 28029 Madrid, Spain; sheisheiabril@gmail.com.
3 Instituto de Química Médica, Consejo Superior de Investigaciones Científicas (IQM-CSIC), 28006 Madrid, Spain
4 Unidad de Química Analítica, Departamento de Química en Ciencias Farmacéuticas. Facultad de Farmacia, Universidad Complutense, 28040 Madrid, Spain; aiolives@ucm.es, mantonia@ucm.es

* Correspondence: rafael.leon@iqm.csic.es (R.L.), josecm@ucm.es (J.C.M.)

Table S1: Predicted physicochemical / lipophilicity / drug-likeness properties for compounds **3**.

**Property**	**3a**	**3b**	**3c**	**3d**	**3e**	**3f**	**3g**	**3h**
MW (g/mol)	333.38	363.41	367.83	391.42	407.46	423.46	437.48	393.43
Nr. heavy atoms	25	27	26	29	30	31	32	29
Nr. ar. heavy atoms	12	12	12	12	12	12	12	12
Fraction Csp^3	0.14	0.18	0.14	0.22	0.25	0.25	0.28	0.22
Nr. rotatable bonds	5	6	5	6	8	8	9	7
Nr. H-bond acceptors	3	4	3	5	5	6	6	5
Nr. H-bond donors	0	0	0	0	0	1	0	1
TPSA (Å^2)	46.61	55.84	46.61	65.07	65.07	85.30	74.30	76.07
Consensus Log $P_{o/w}$	3.30	3.29	3.82	3.44	3.61	3.01	3.56	3.23
Lipinski violations	0	0	0	0	0	0	0	0
PAINS	1	1	1	1	1	1	1	1

**Property**	**3i**	**3j**	**3k**	**3l**	**3m**	**3n**	**3o**
MW (g/mol)	379.41	501.55	518.00	518.00	578.05	578.05	386.44
Nr. heavy atoms	28	37	37	37	41	41	29
Nr. ar. heavy atoms	12	18	18	18	18	18	15
Fraction Csp^3	0.18	0.20	0.20	0.20	0.25	0.25	0.17
Nr. rotatable bonds	6	10	10	10	12	12	6
Nr. H-bond acceptors	5	6	5	5	7	7	3
Nr. H-bond donors	2	0	0	0	0	0	1
TPSA (Å^2)	87.07	65.07	65.07	65.07	83.53	83.53	62.40
Consensus Log $P_{o/w}$	2.82	5.06	5.31	5.30	5.29	5.24	3.71
Lipinski violations	0	1	1	1	1	1	0
PAINS	2	1	1	1	1	1	1

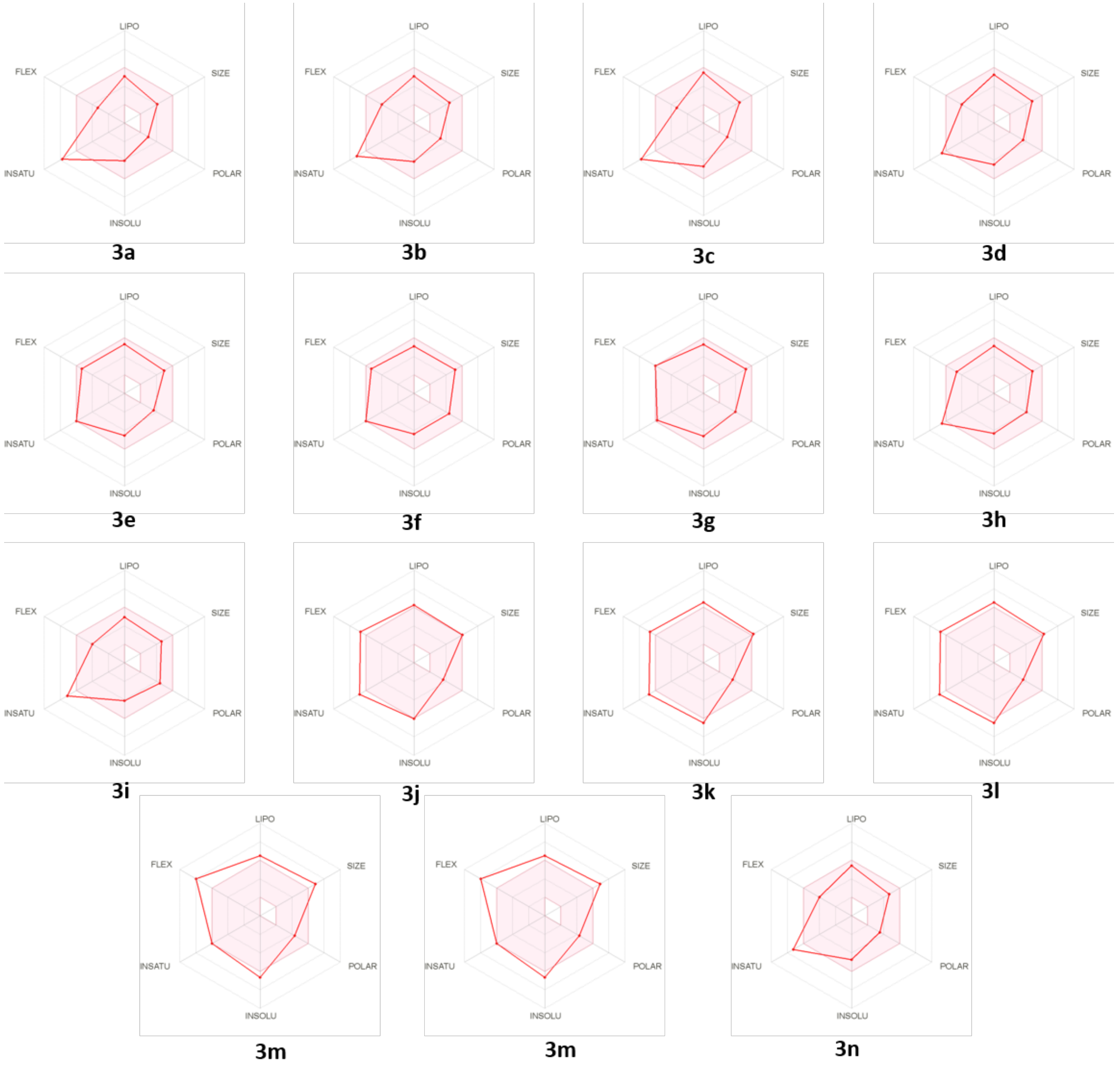

Figure S1: Bioavailability radar of compounds **3** in colored zones delimitated by physicochemical indices that are considered ideal for oral bioavailability.[1] LIPO, Lipophilicity: -0.7 < XLOGP3 < 5; SIZE, Molecular size: 150 g/mol < mol.wt. < 500 g/mol; POLAR, Polarity: 20 Å^2 < TPSA < 130 Å^2; INSOLU, Insolubility: 0 < Log S (ESOL) < 6; INSATU, Insaturation: 0.25 < Fraction Csp^3 < 1; FLEX, Flexibility: 0 < Number of rotatable bonds < 9.

[1] Daina, A.; Michielin, O.; Zoete, V. SwissADME: a free web tool to evaluate pharmacokinetics, drug-likeness and medicinal chemistry friendliness of small molecules. *Sci. Rep.* **2017**, *7*, 42717.

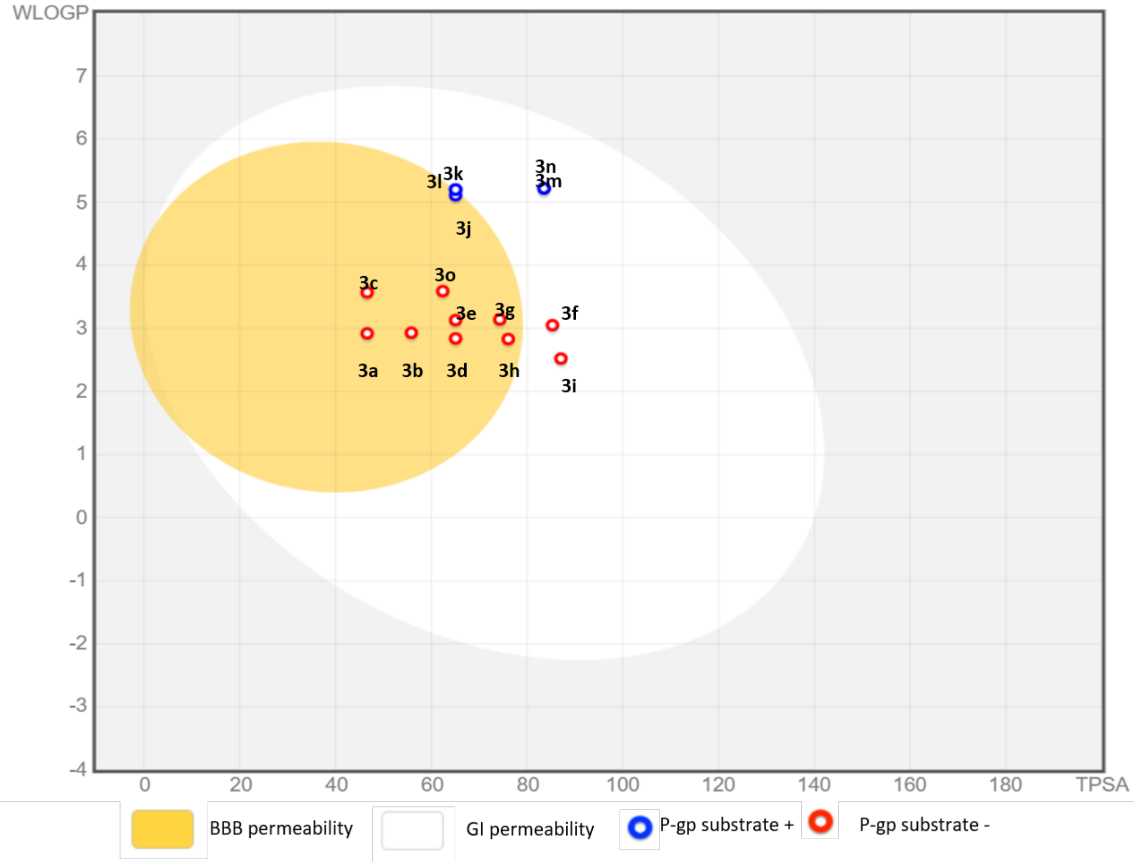

Figure S2. Boiled-egg model of identified compounds for the prediction of gastrointestinal and blood-brain barrier permeability.[2] WLOGP: log *P* calculation according to Wildman and Crippen.

[2] Daina, A.; Zoete, V. A BOILED-egg to predict gastrointestinal absorption and brain penetration of small molecules. *ChemMedChem* **2016**, *11*, 1117–1121.

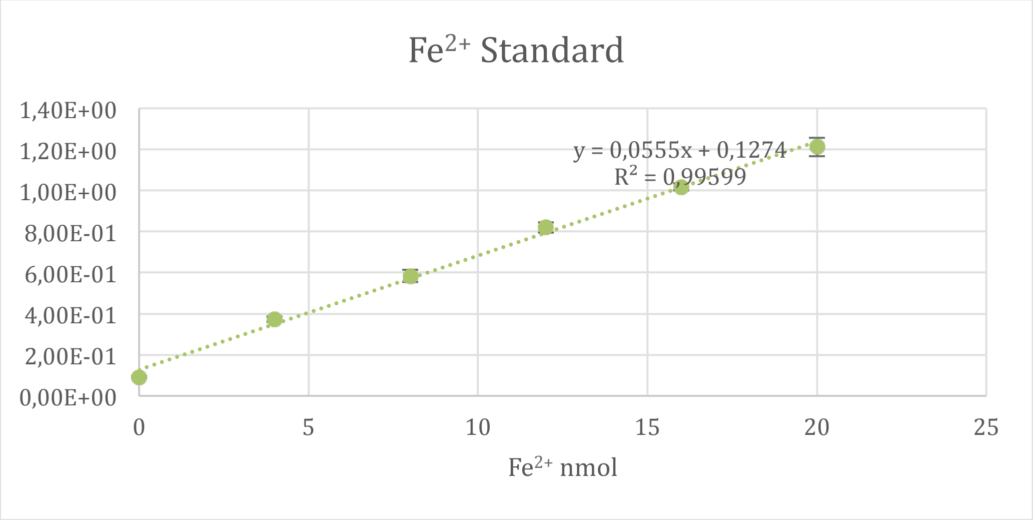

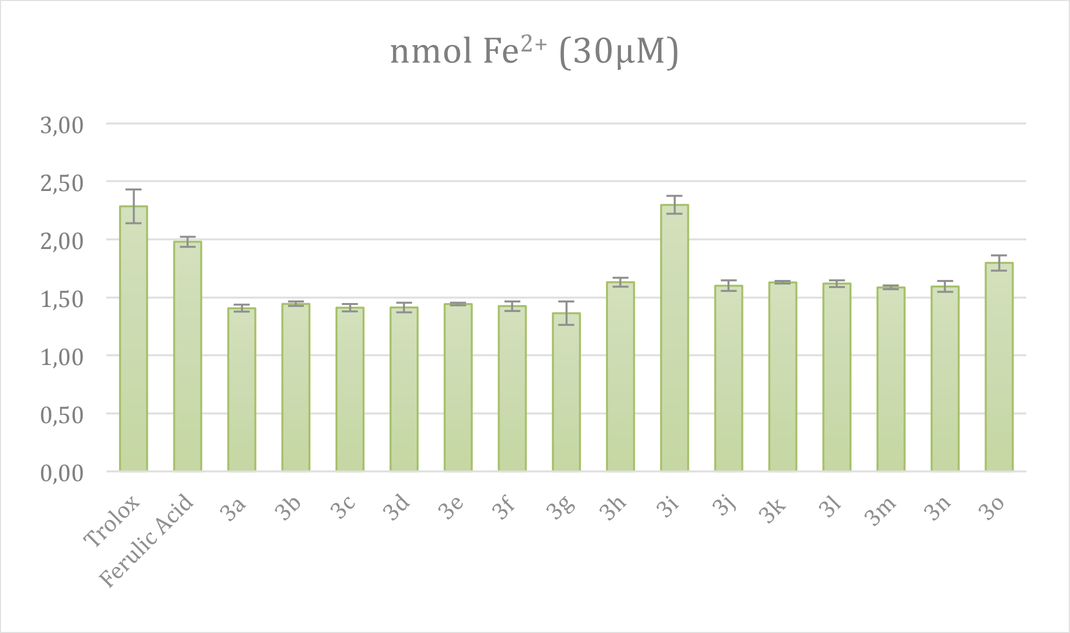

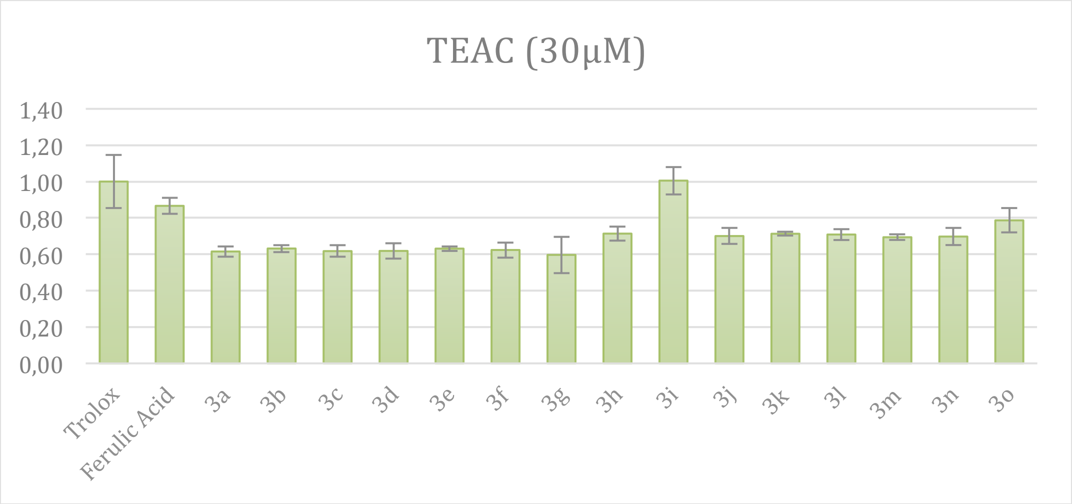

Figure S3. Antioxidant activity of compounds **3** by the ferric reducing antioxidant power (FRAP) method

Table S2. Neuroprotection elicited by compounds **3** in SH-SY5Y cells following treatment with rotenone (30 μM) and olygomicin A (10 μM) [a]

Entry	Compound	% Neuroprotection ± SEM at 1 μM	Statistical significance
1	**Basal**	100	
2	**R/O (30/10 μM)**		###
3	**Melatonin**	61.41 ± 8.75	***
4	**3a**	44.33 ± 13.57	**
5	**3b**	68.05 ± 31.51	*
6	**3c**	94.91 ± 24.25	***
7	**3d**	49.79 ± 9.46	**
8	**3e**	50.32 ± 16.34	**
9	**3f**	50.99 ± 16.93	**
10	**3g**	74.47 ± 7.19	*
11	**3h**	46.23 ± 18.89	**
12	**3i**	90.40 ± 32.38	**
13	**3j**	51.19 ± 15.49	**
14	**3k**	60.26 ± 20.86	**
15	**3l**	79.76 ± 8.28	*
16	**3m**	41.18 ± 18.85	*
17	**3n**	69.40 ± 23.95	**
18	**3o**	90.12 ± 8.80	***

[a] Data are expressed as mean ± SEM of three experiments by triplicate. One way ANOVA Newman Keuls post test ### $p < 0.001$; compared to basal. * $p < 0.05$, ** $p < 0.01$; *** $p < 0.001$; compared to toxic.

Table S3. Neuroprotection elicited by compounds **3** in SH-SY5Y cells following treatment with okadaic acid (20 nM)

Entry	Compound	% Neuroprotection ± SEM at 1 μM	Statistical significance
1	**Basal**		
2	**OA (20 nM)**		###
3	**Melatonin**	67.70 ± 13.95	**
4	**3a**	68.05 ± 22.01	*
5	**3b**	54.15 ± 14.26	**
6	**3c**	52.77 ± 9.96	**
7	**3d**	52.71 ± 17.20	*
8	**3e**	62.77 ± 8.00	**
9	**3f**	48.30 ± 10.99	**
10	**3g**	65.73 ± 29.07	**
11	**3h**	81.86 ± 28.54	*
12	**3i**	60.87 ± 23.33	**
13	**3j**	31.82 ± 15.21	
14	**3k**	57.14 ± 22.31	*
15	**3l**	51.07 ± 18.36	**
16	**3m**	40.72 ± 9.85	*
17	**3n**	61.39 ± 15.75	**
18	**3o**	46.27 ± 13.57	*

[a] Data are expressed as mean ± SEM of three experiments by triplicate. One way ANOVA Newman Keuls post test ### $p < 0.001$; compared to basal. * $p < 0.05$, ** $p < 0.01$; *** $p < 0.001$; compared to toxic.

Copies of spectra

4-[(2-Fluorobenzyl)oxy]-3-methoxybenzaldehyde (**2a**)

^{1}H NMR (250 MHz, $CDCl_3$)

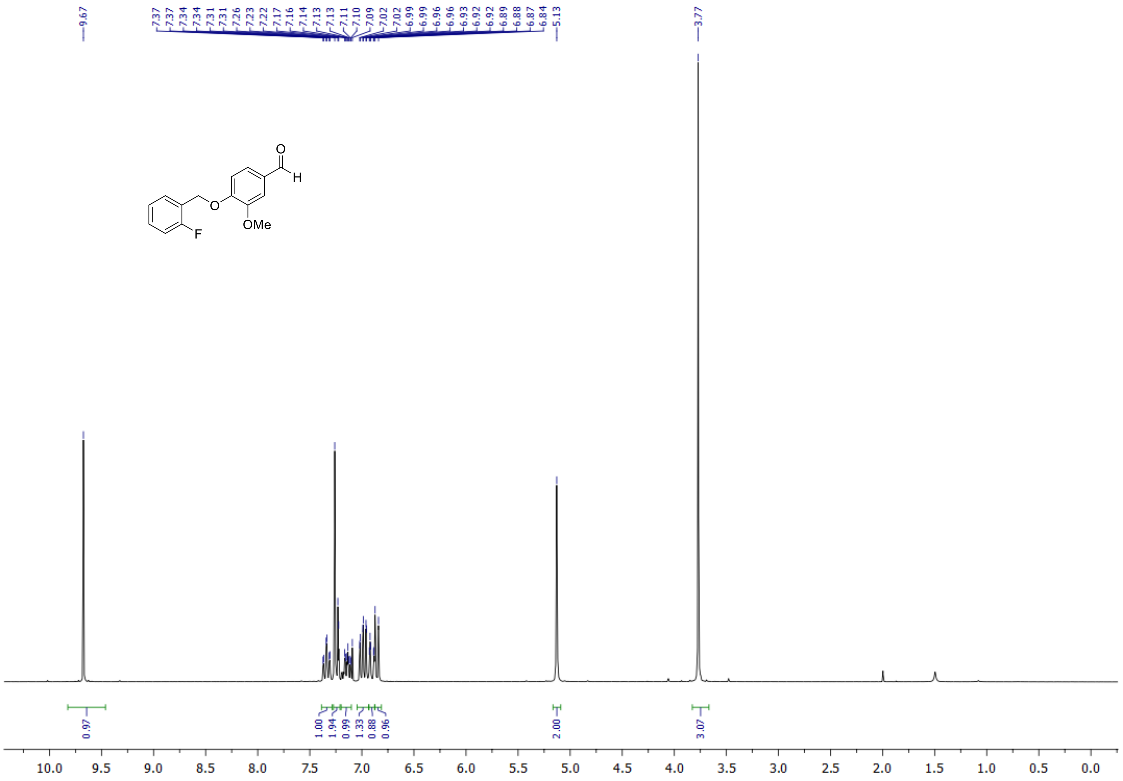

^{13}C NMR (63 MHz, $CDCl_3$)

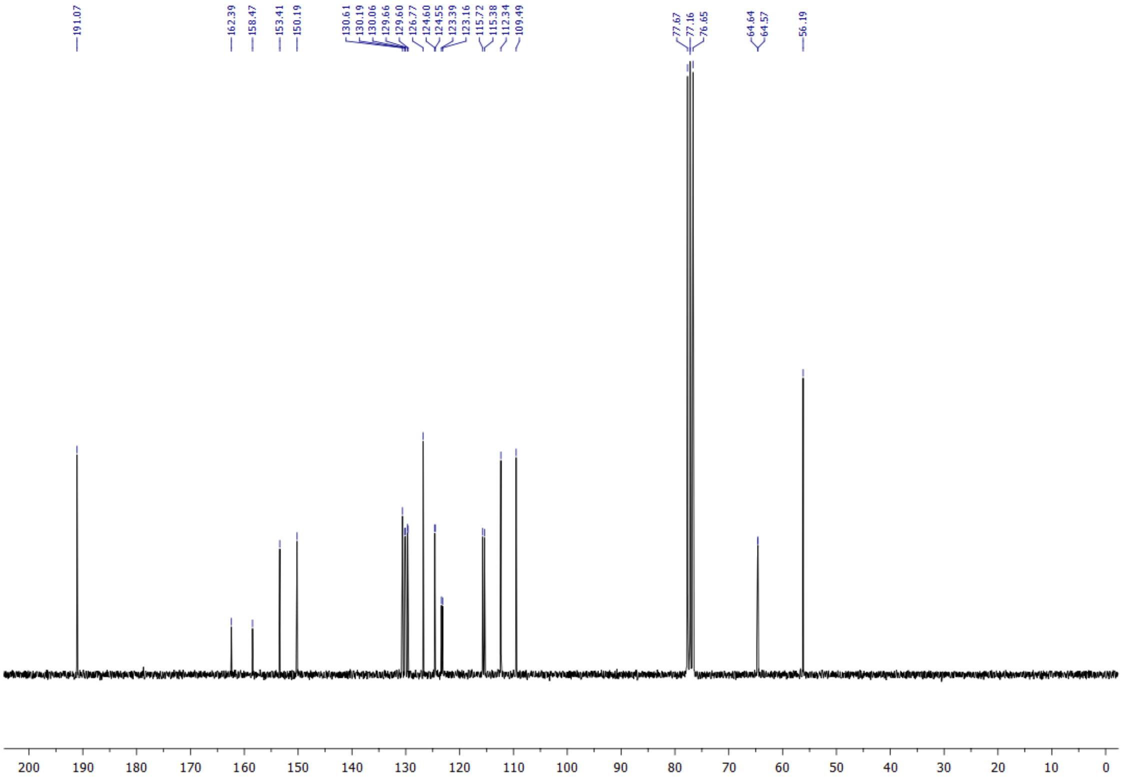

4-[(2-Chlorobenzyl)oxy]-3-methoxybenzaldehyde (**2b**)

^{1}H NMR (250 MHz, $CDCl_3$)

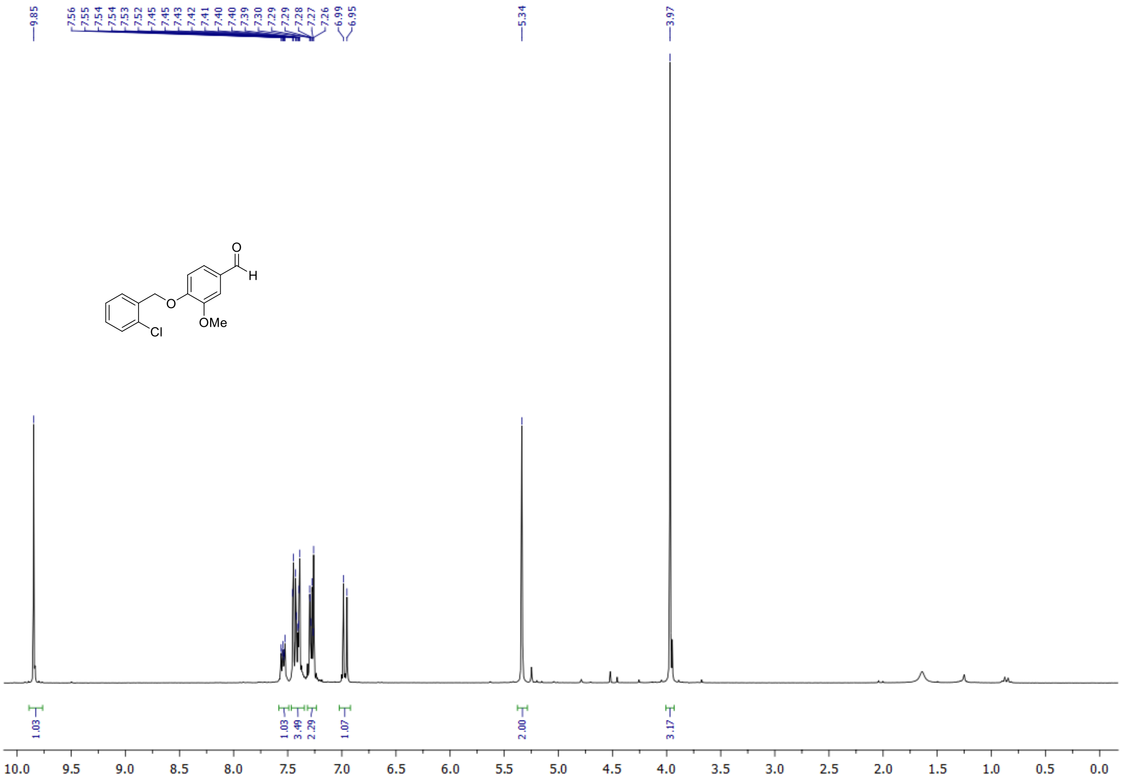

^{13}C NMR (63 MHz, $CDCl_3$)

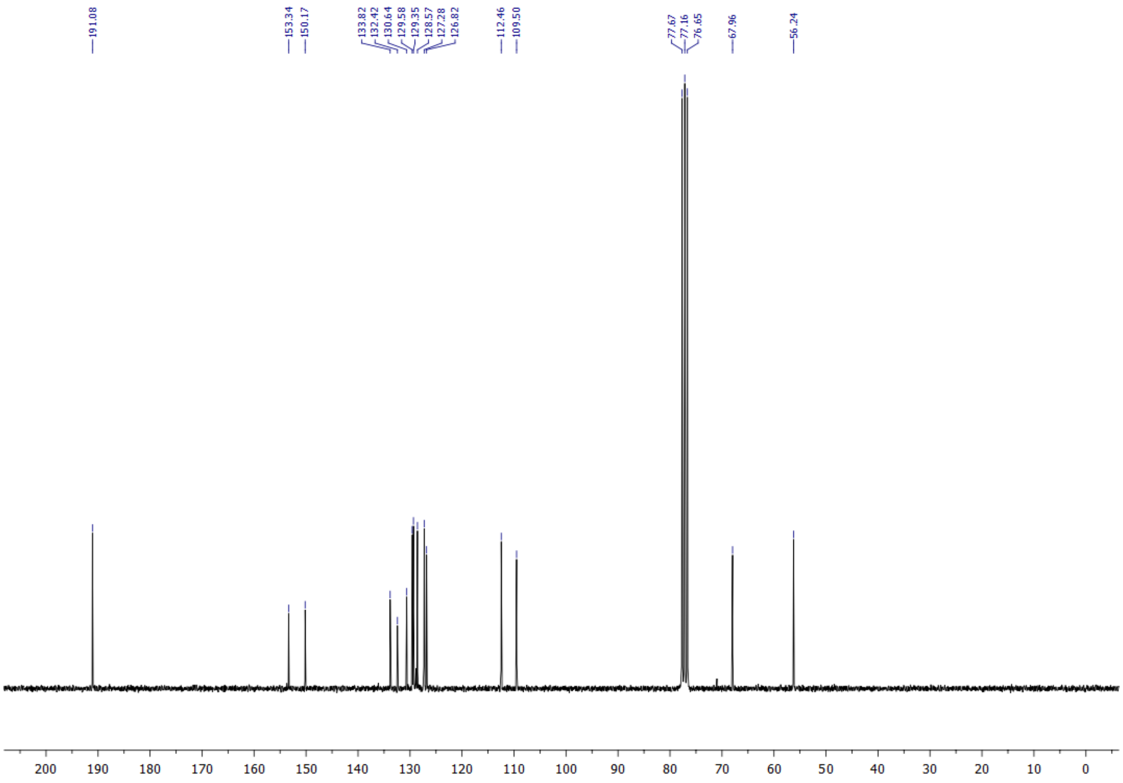

4-[(4-Chlorobenzyl)oxy]-3-methoxybenzaldehyde (**2c**)

^{1}H NMR (250 MHz, $CDCl_3$)

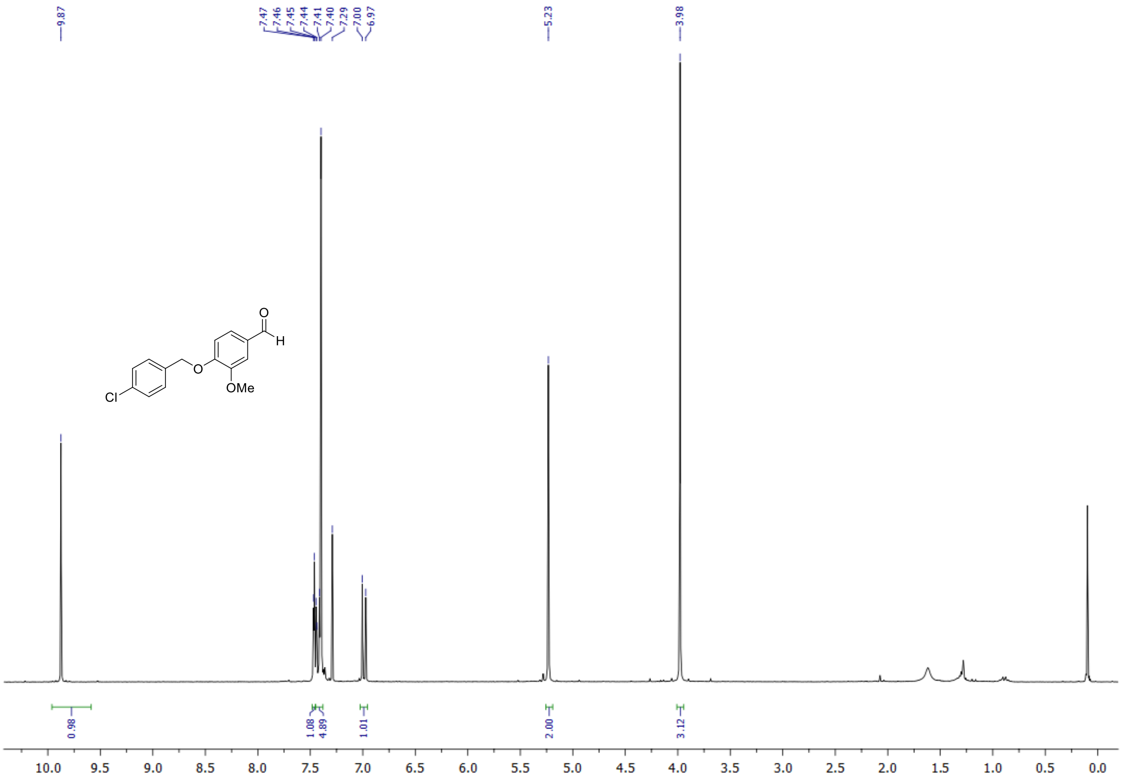

^{13}C NMR (63 MHz, $CDCl_3$)

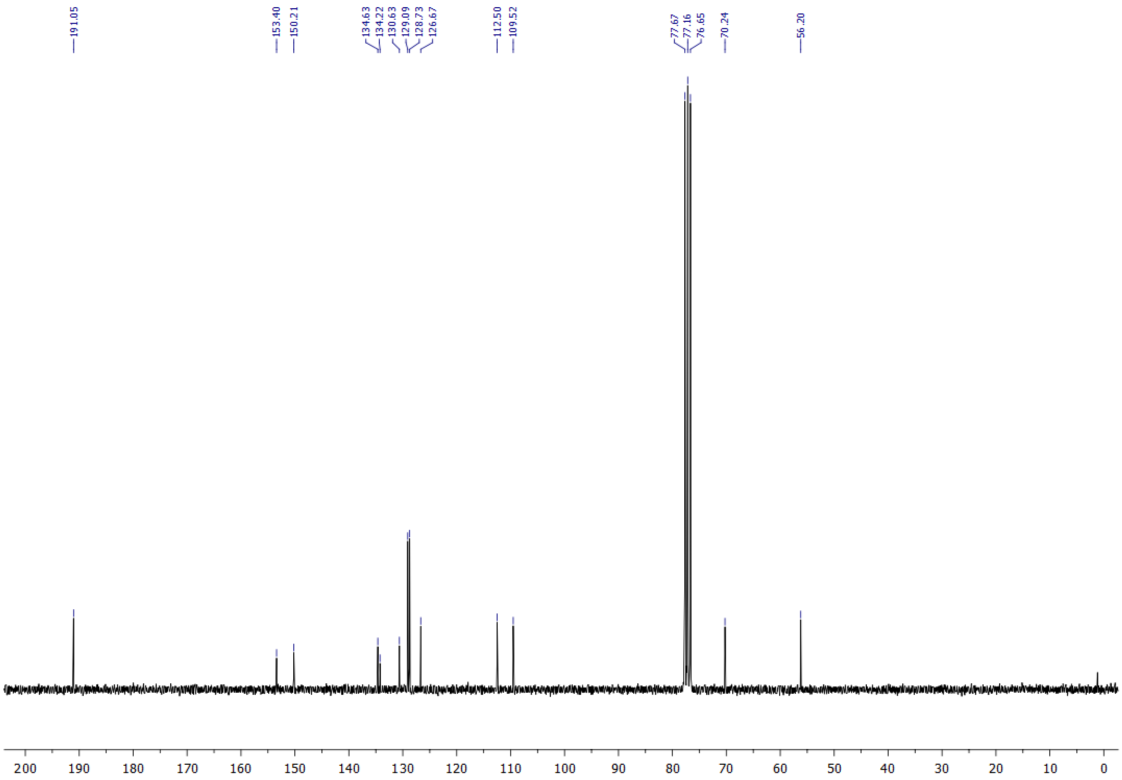

Methyl (*Z*)-1-benzyl-4-benzylidene-2-methyl-5-oxo-4,5-dihydro-1*H*-pyrrole-3-carboxylate (**3a**)

^{1}H NMR (250 MHz, $CDCl_3$)

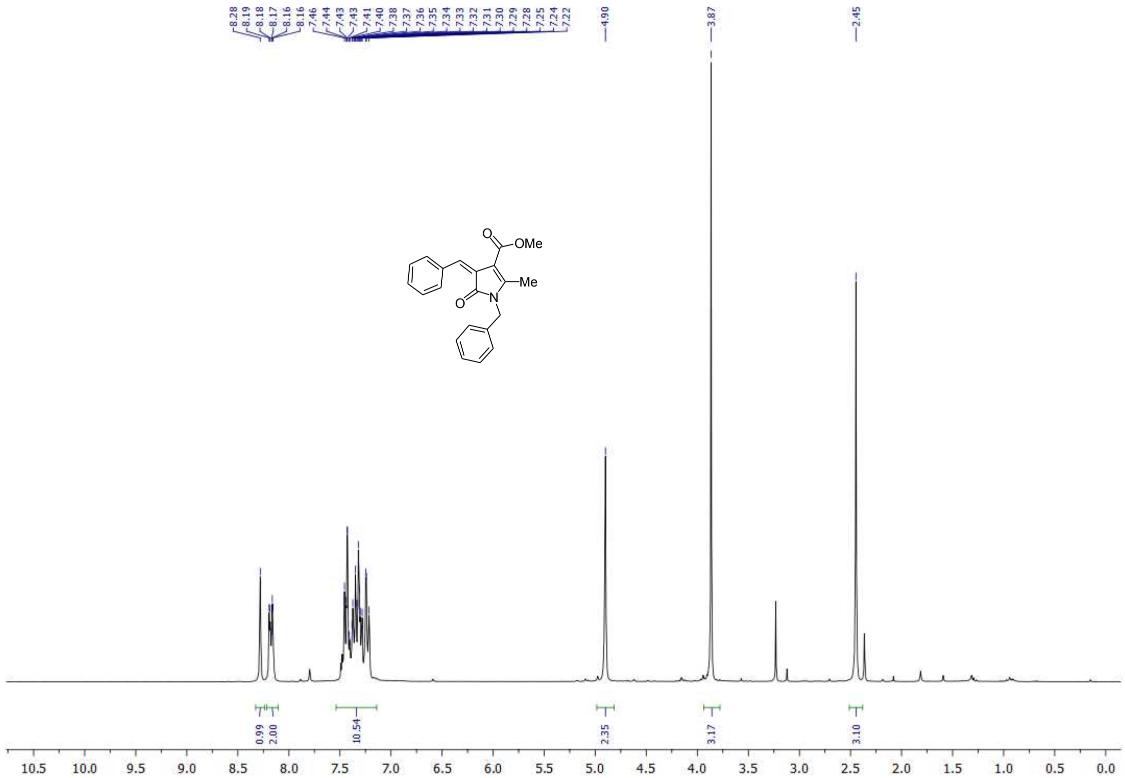

^{13}C NMR (63 MHz, $CDCl_3$)

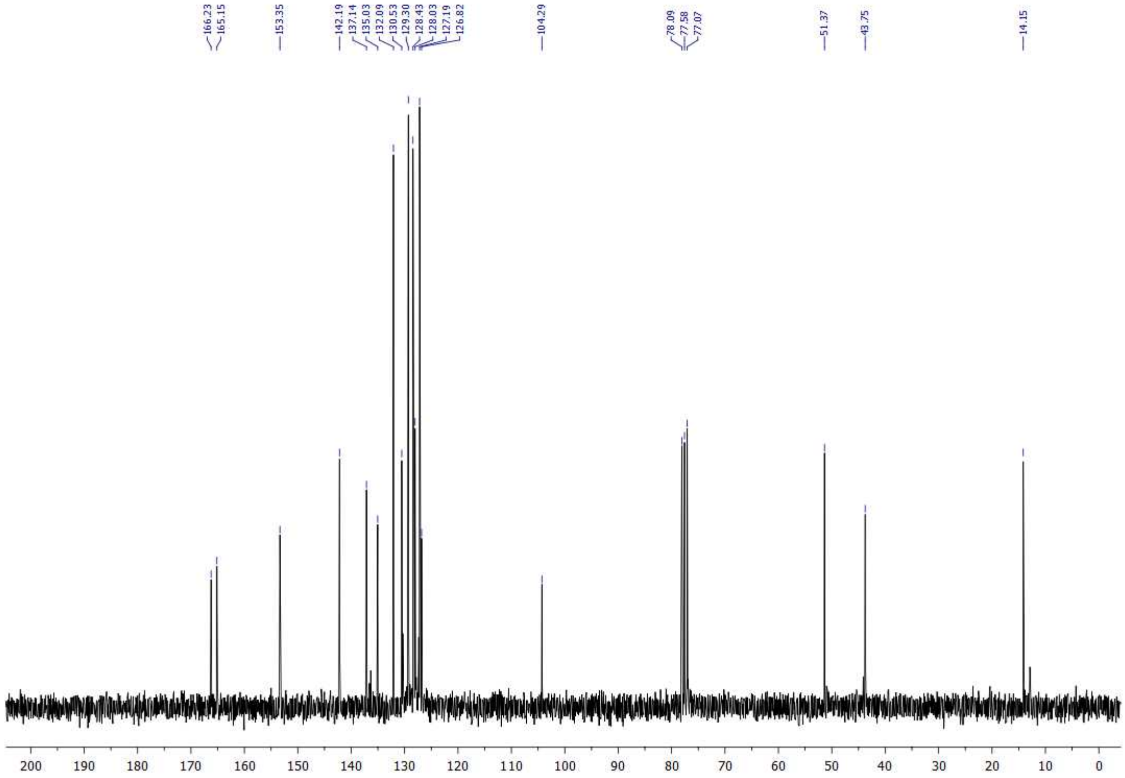

Methyl (*Z*)-1-benzyl-4-(4-methoxybenzylidene)-2-methyl-5-oxo-4,5-dihydro-1*H*-pyrrole-3- carboxylate (**3b**)

^{1}H NMR (250 MHz, $CDCl_3$)

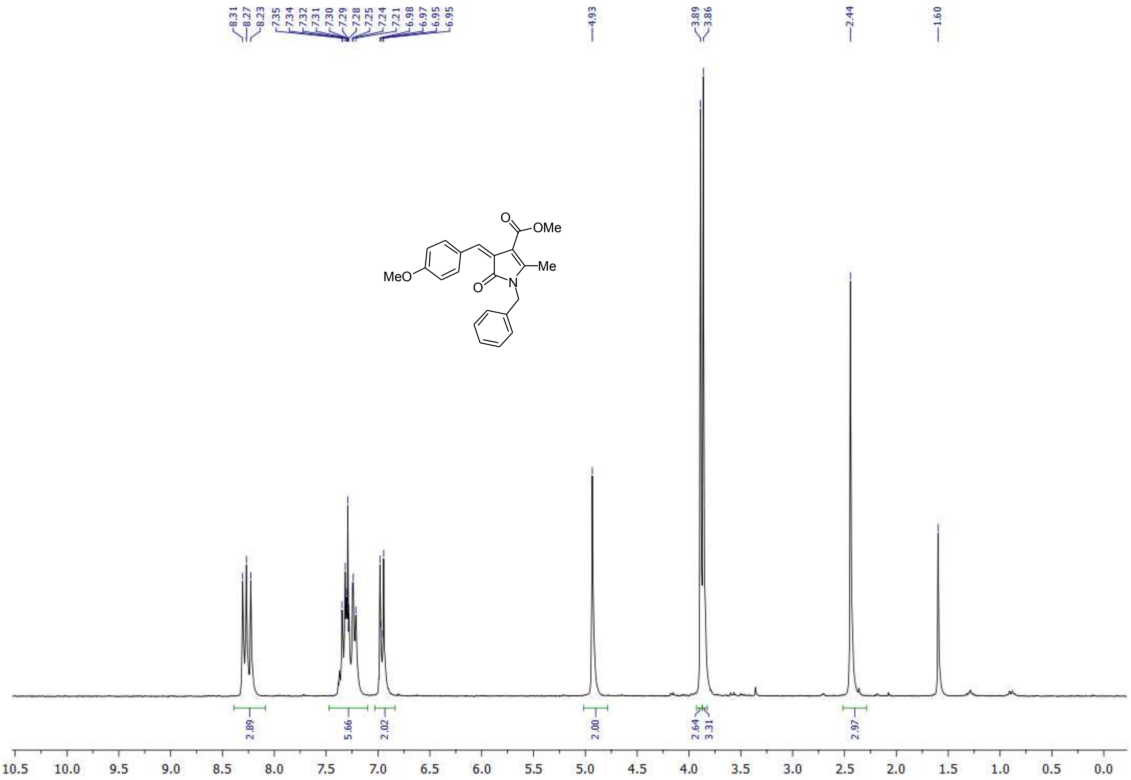

^{13}C NMR (63 MHz, $CDCl_3$)

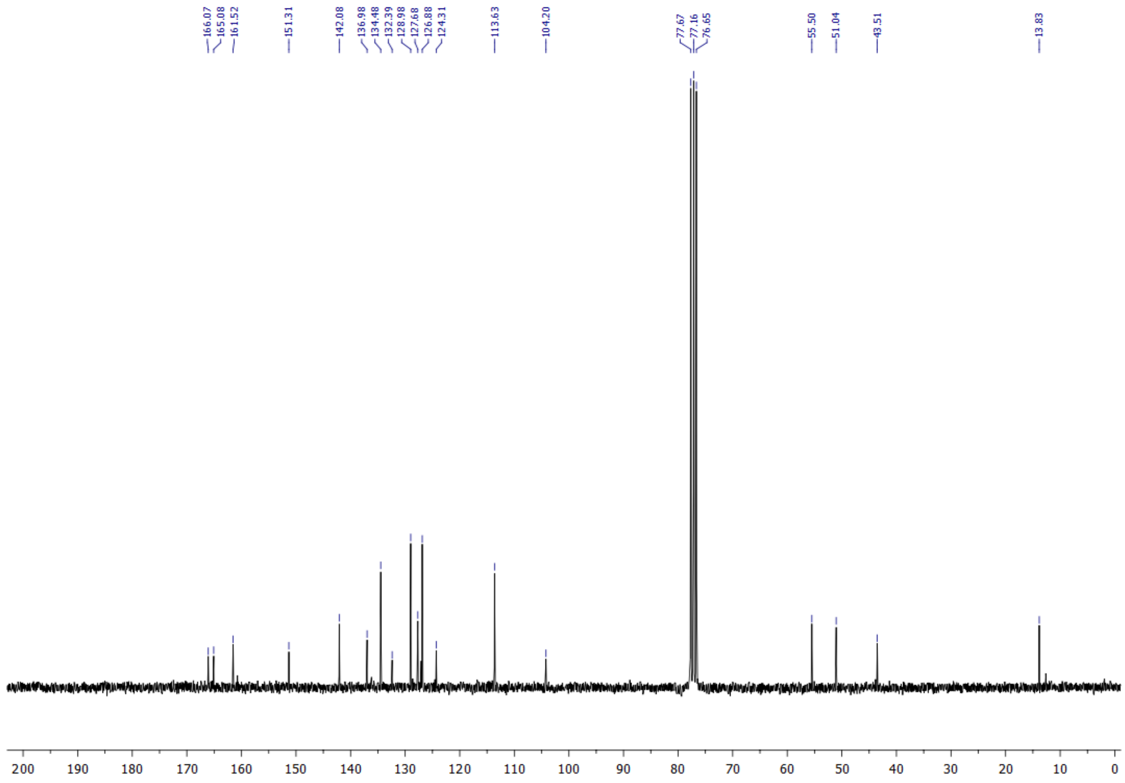

Methyl (*Z*)-1-benzyl-4-(4-chlorobenzylidene)-2-methyl-5-oxo-4,5-dihydro-1*H*-pyrrole-3- carboxylate (**3c**)

^{1}H NMR (250 MHz, CDCl$_3$)

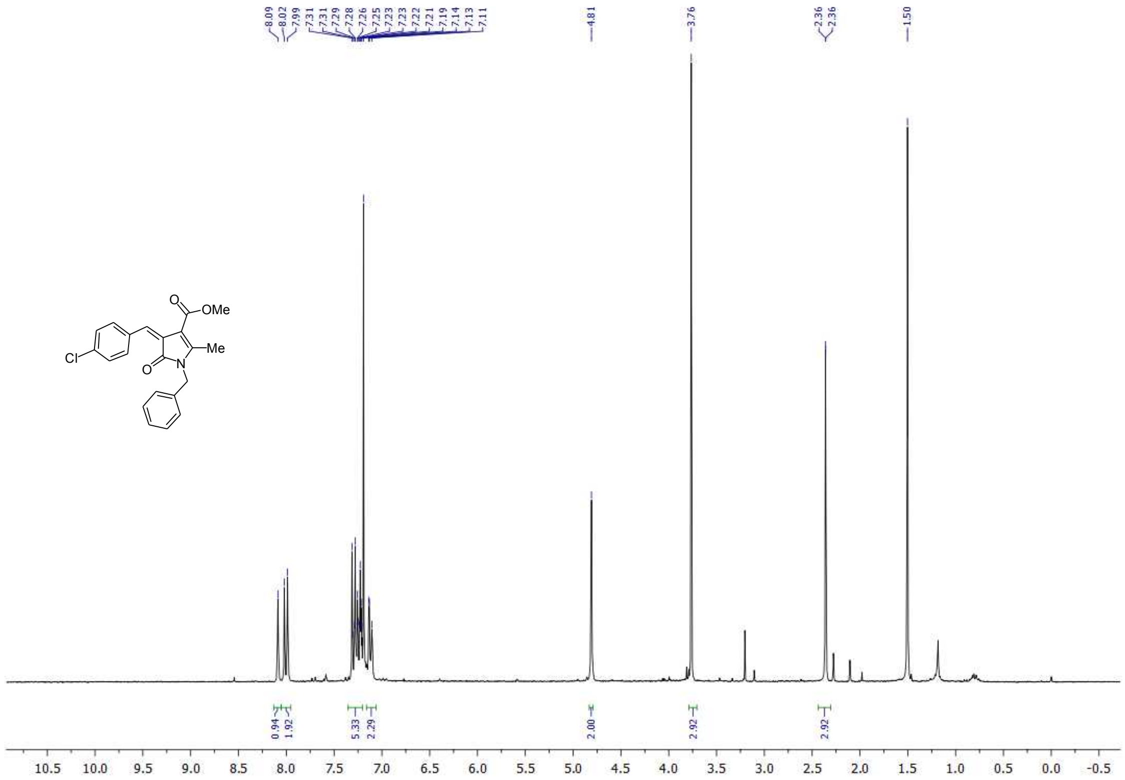

^{13}C NMR (63 MHz, CDCl$_3$)

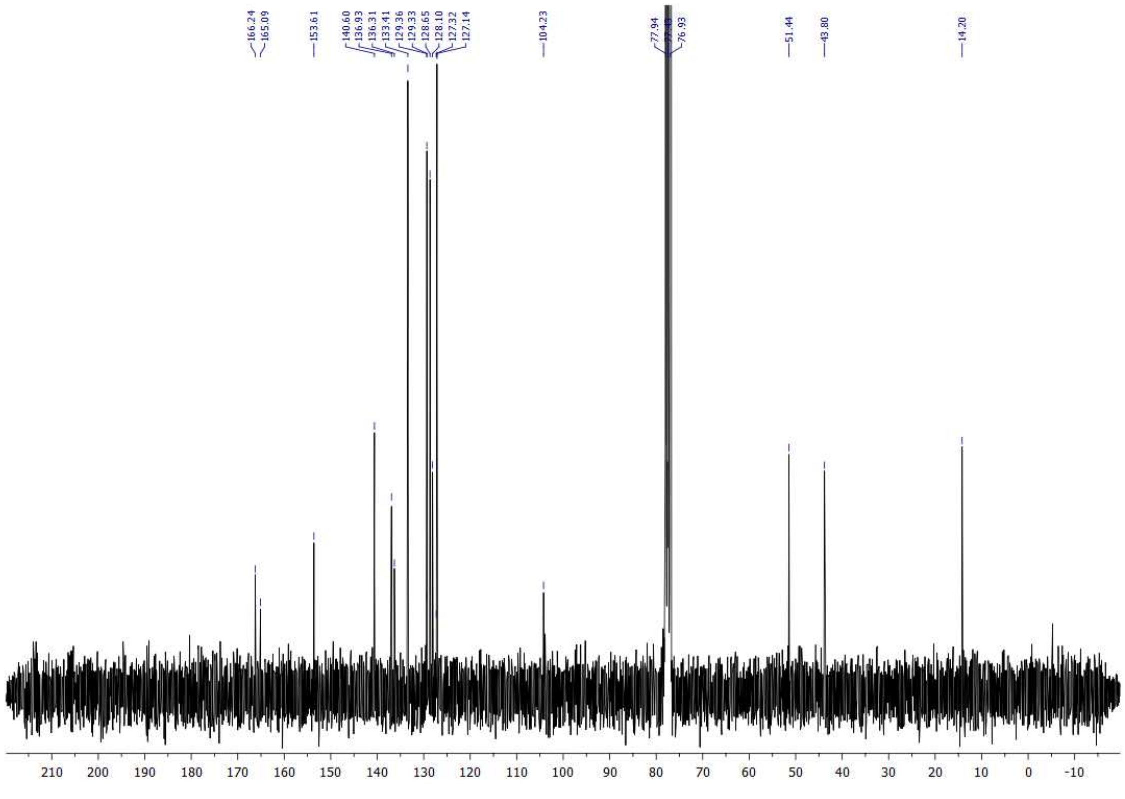

Methyl (*Z*)-4-(benzo[d][1,3]dioxol-5-ylmethylene)-2-methyl-5-oxo-1-phenethyl-4,5-dihydro-1*H*-pyrrole-3-carboxylate (**3d**)

^{1}H NMR (250 MHz, CDCl$_3$)

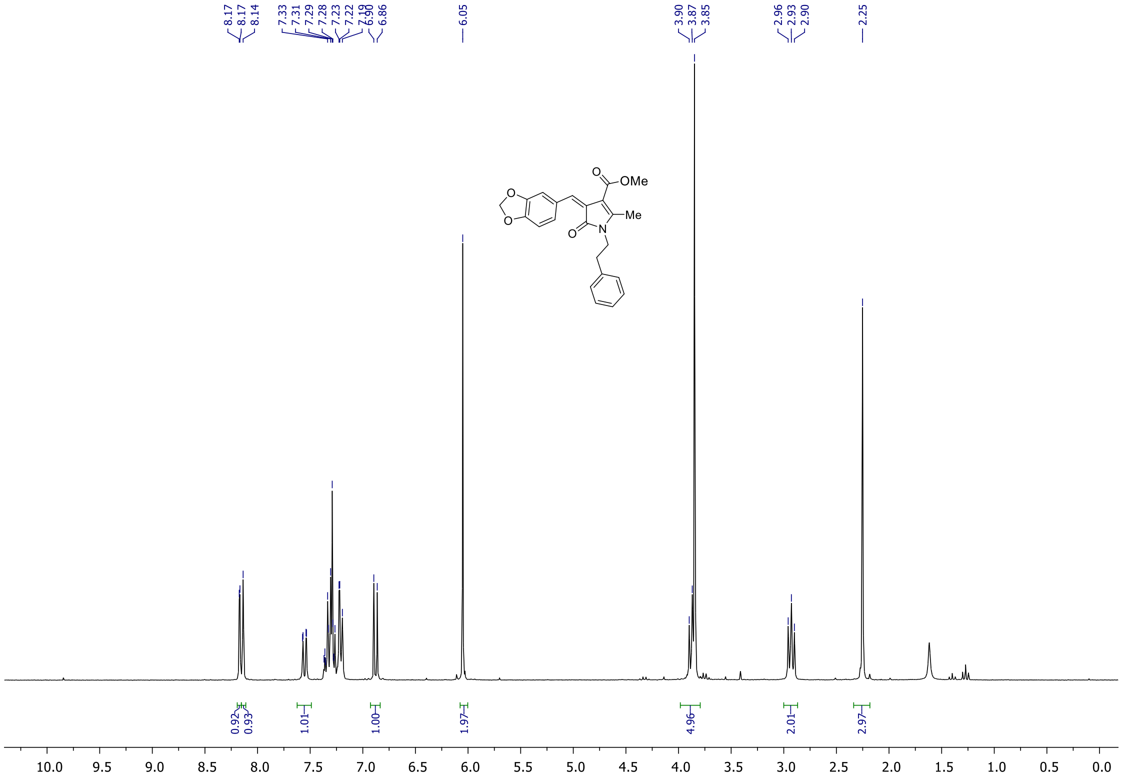

^{13}C NMR (63 MHz, CDCl$_3$)

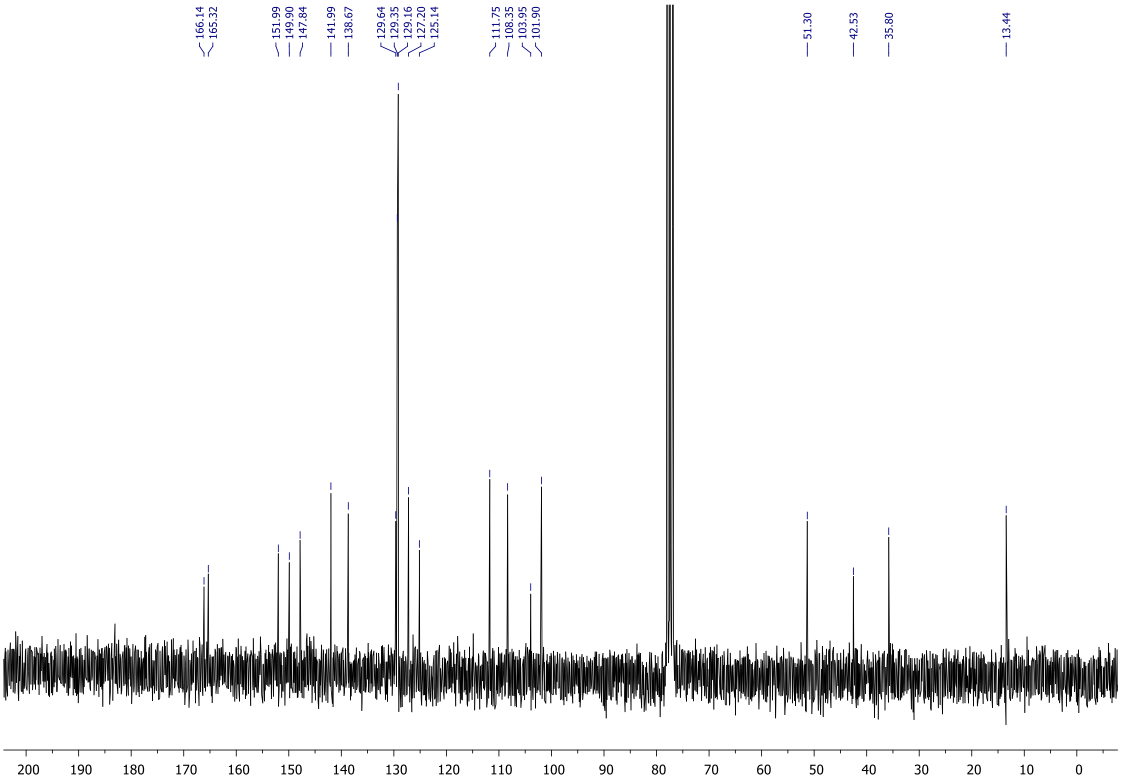

Methyl (*Z*)-4-(2,4-dimethoxybenzylidene)-2-methyl-5-oxo-1-phenethyl-4,5-dihydro-1*H*-pyrrole-3-carboxylate (**3e**)

^{1}H NMR (250 MHz, $CDCl_3$)

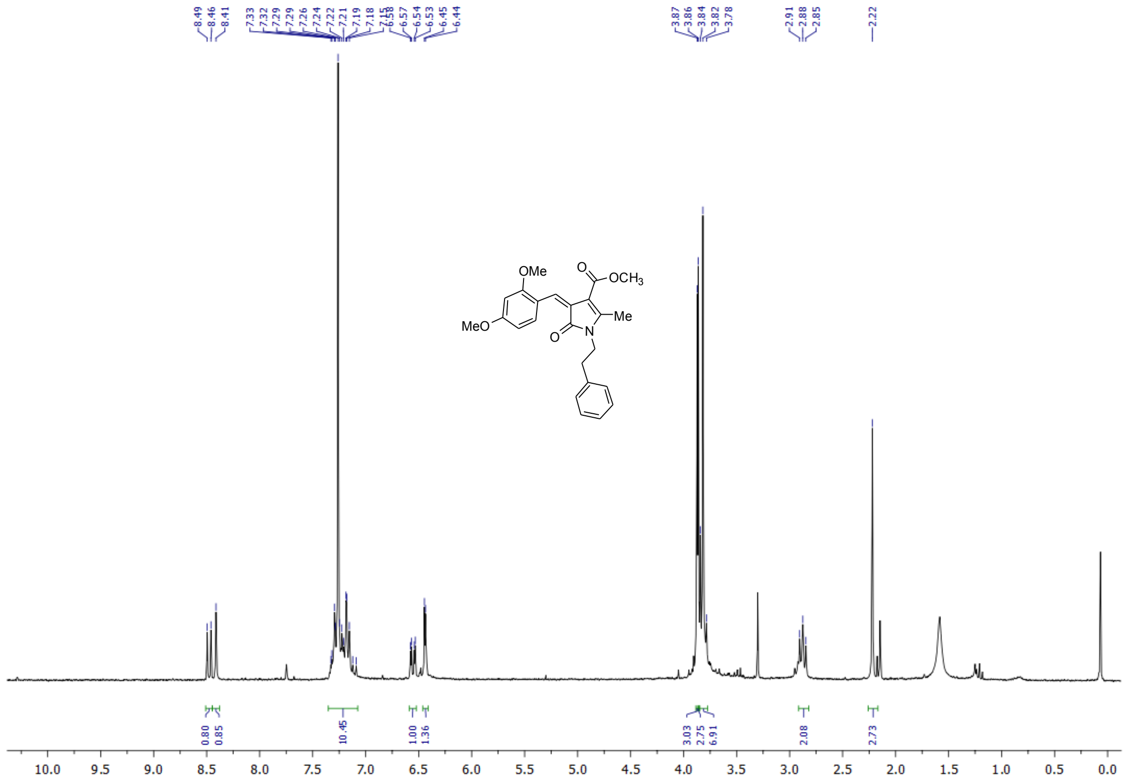

^{13}C NMR (63 MHz, $CDCl_3$)

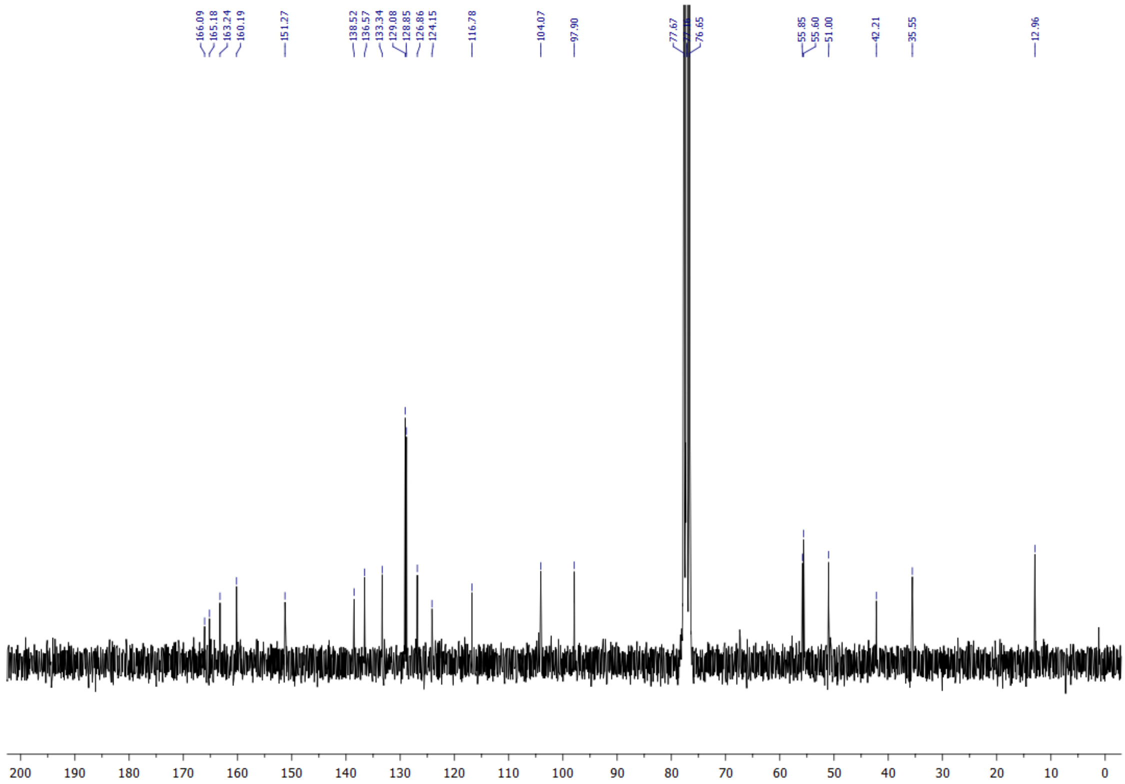

Methyl (*Z*)-2-methyl-5-oxo-1-phenethyl-4-(2,4,5-trimethoxybenzylidene)-4,5-dihydro-1*H*- pyrrole-3-carboxylate (**3f**)

^{1}H NMR (250 MHz, $CDCl_3$)

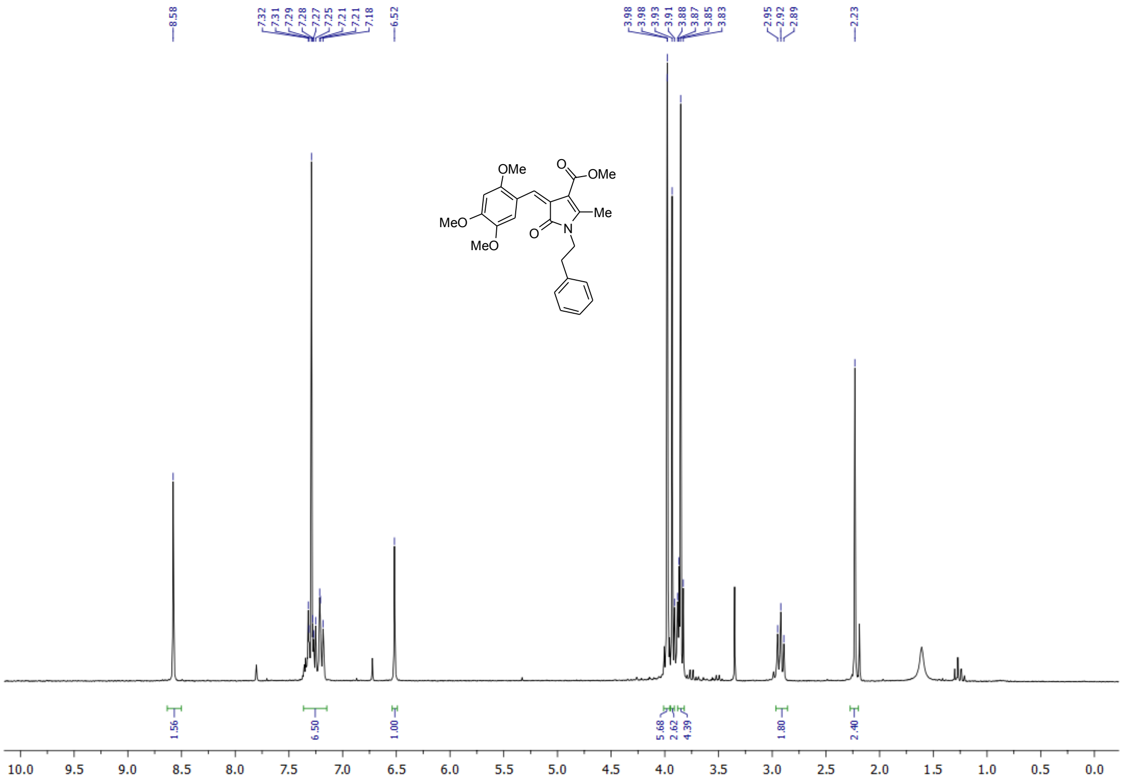

^{13}C NMR (63 MHz, $CDCl_3$)

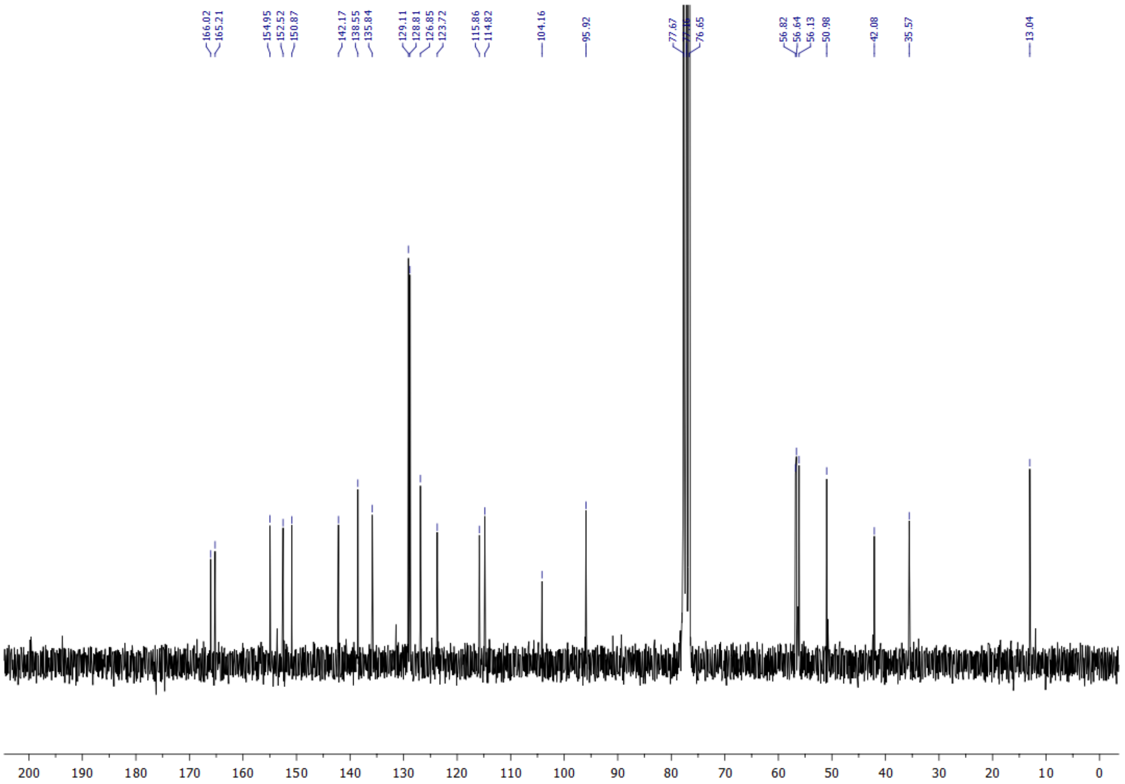

Methyl (*Z*)-2-methyl-5-oxo-1-phenethyl-4-(3,4,5-trimethoxybenzylidene)-4,5-dihydro-1*H*- pyrrole-3-carboxylate (**3g**)

^{1}H NMR (250 MHz, CDCl$_3$)

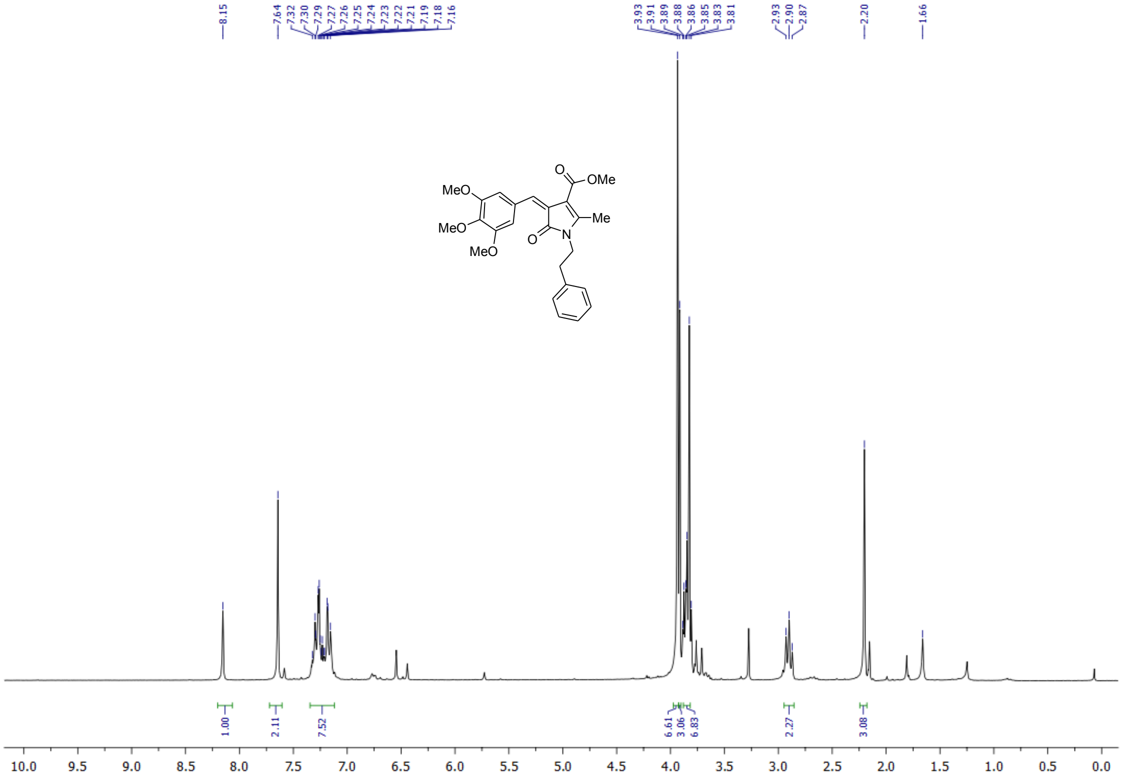

^{13}C NMR (63 MHz, CDCl$_3$)

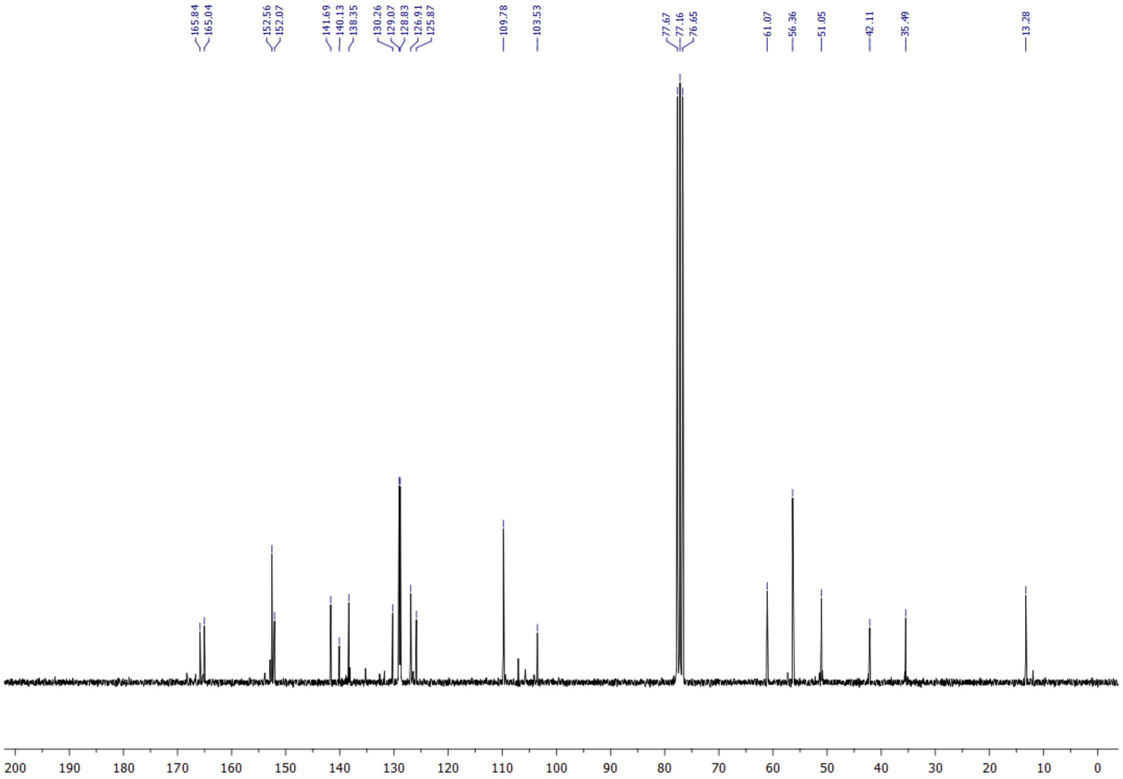

Methyl (*Z*)-4-(3-hydroxy-4-methoxybenzylidene)-2-methyl-5-oxo-1-phenethyl-4,5-dihydro- 1*H*-pyrrole-3- carboxylate (**3h**)

^{1}H NMR (250 MHz, CDCl$_3$)

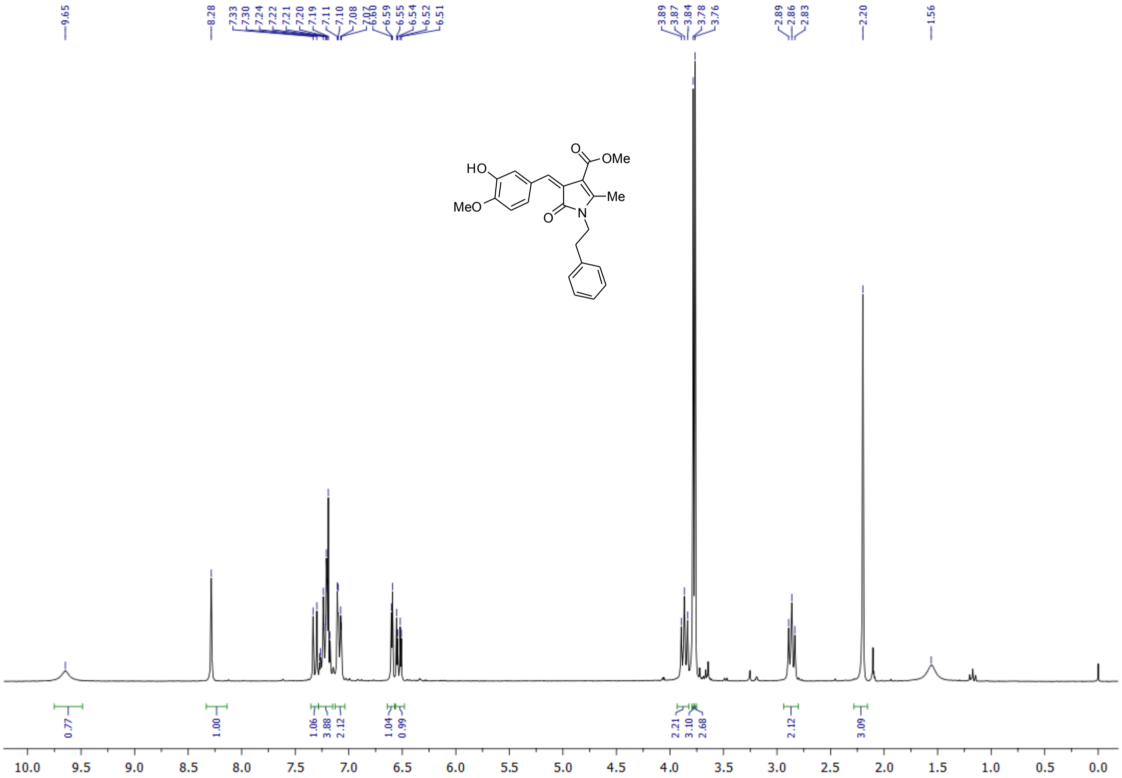

^{13}C NMR (63 MHz, CDCl$_3$)

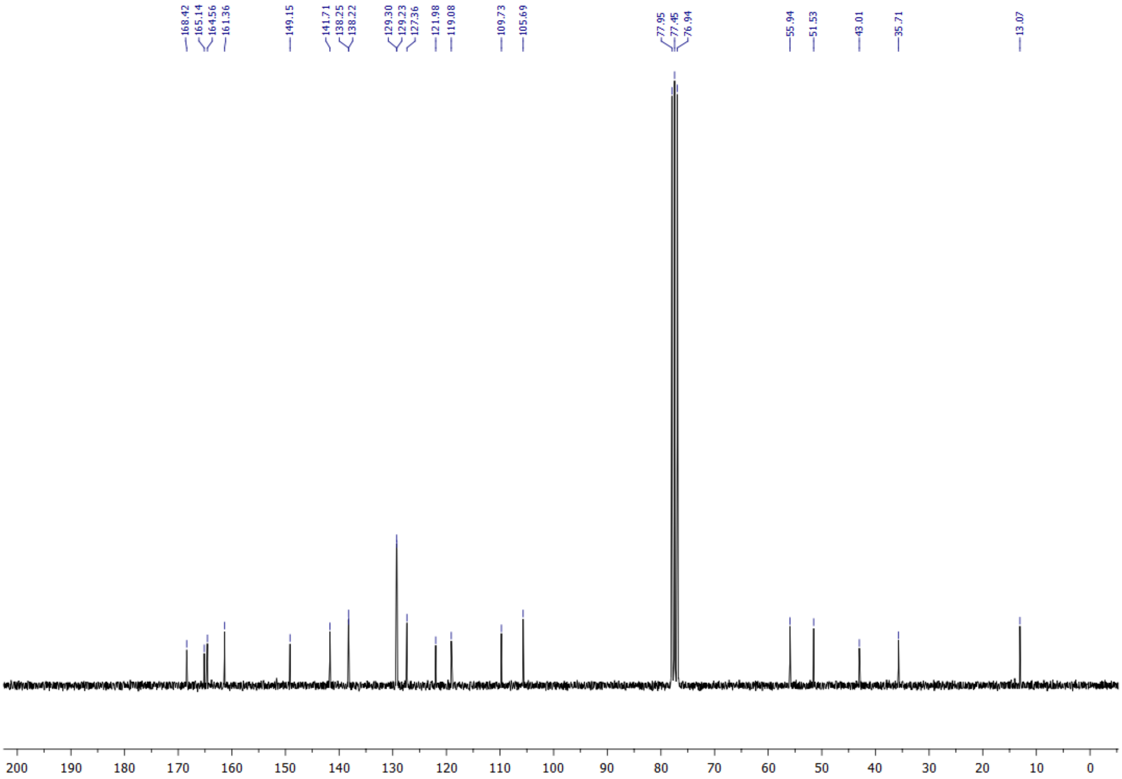

Methyl (*Z*)-4-(3,4-dihydroxybenzylidene)-2-methyl-5-oxo-1-phenethyl-4,5-dihydro-1*H*-pyrrole-3-carboxylate (**3i**)

^{1}H NMR (250 MHz, CDCl$_3$)

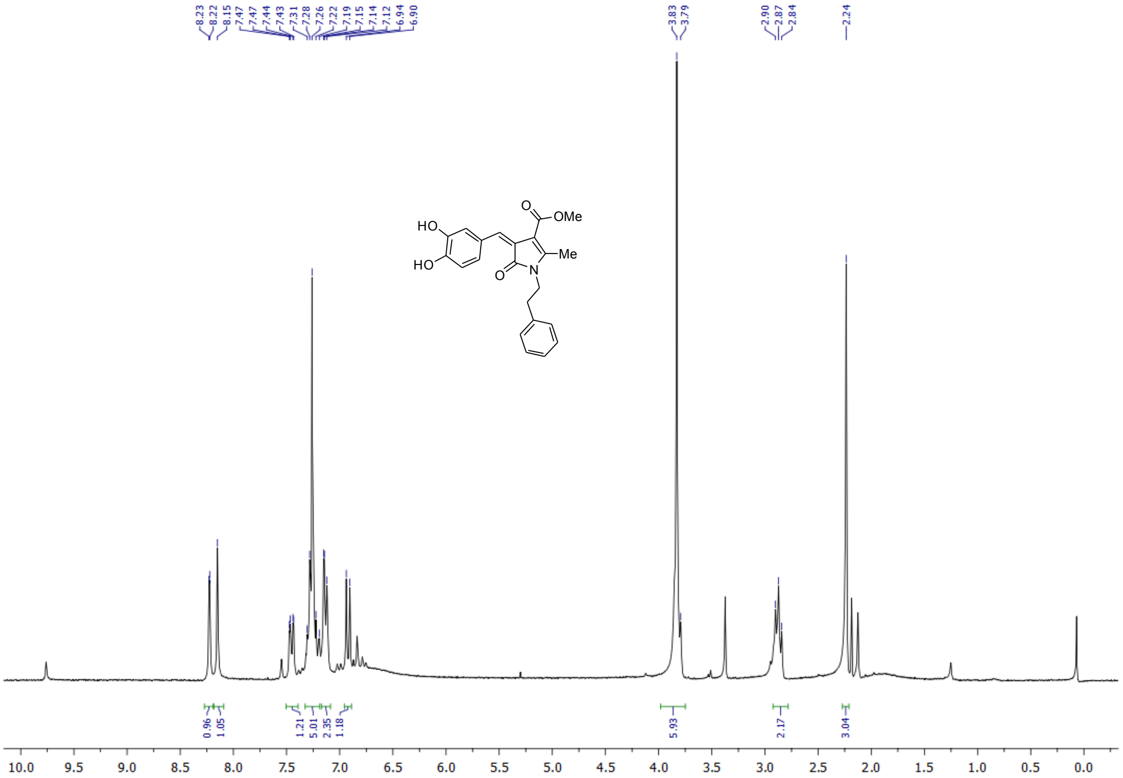

^{13}C NMR (63 MHz, CDCl$_3$)

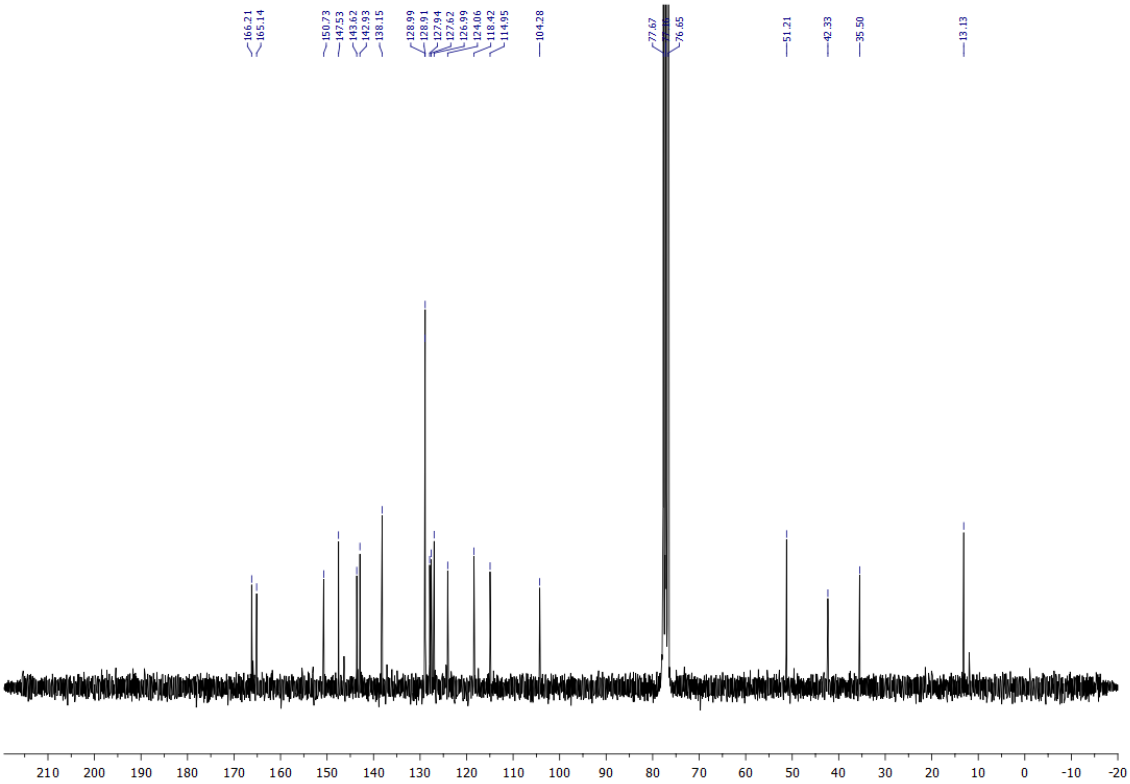

Methyl (*Z*)-4-[4-[(2-fluorobenzyl)oxy]-3-methoxybenzylidene]-2-methyl-5-oxo-1-phenethyl- 4,5-dihydro-1*H*-pyrrole-3-carboxylate (**3j**)

^{1}H NMR (250 MHz, $CDCl_3$)

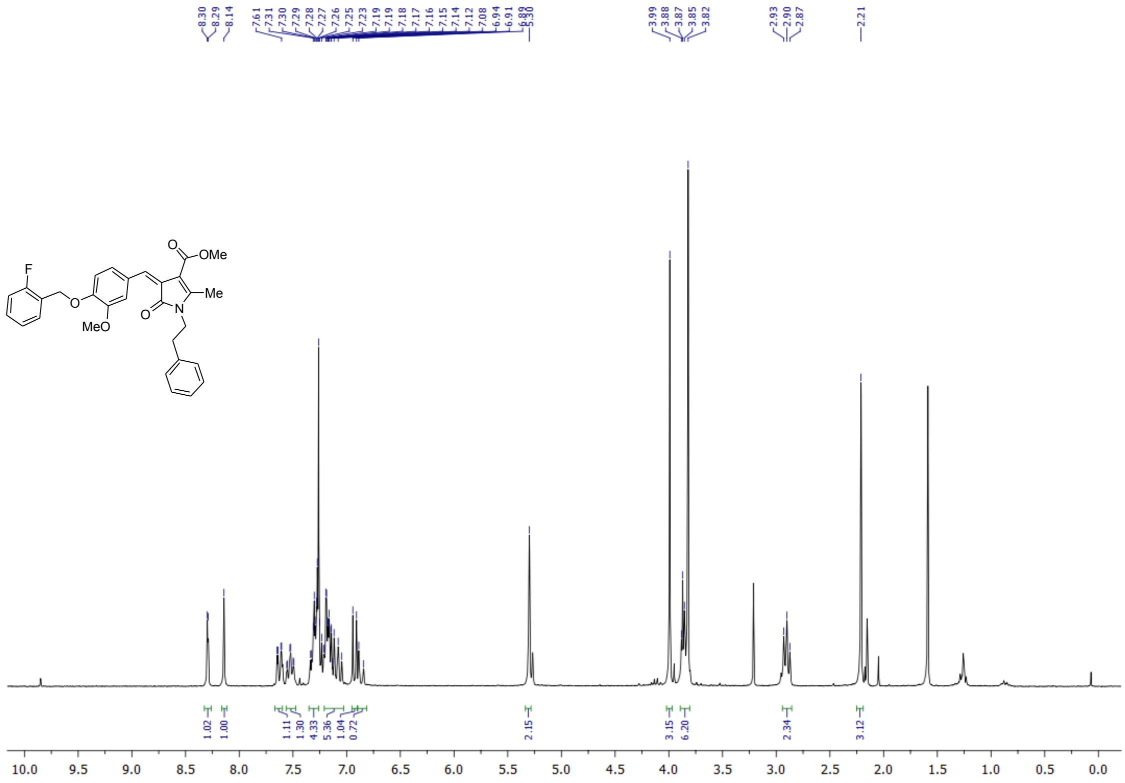

^{13}C NMR (63 MHz, $CDCl_3$)

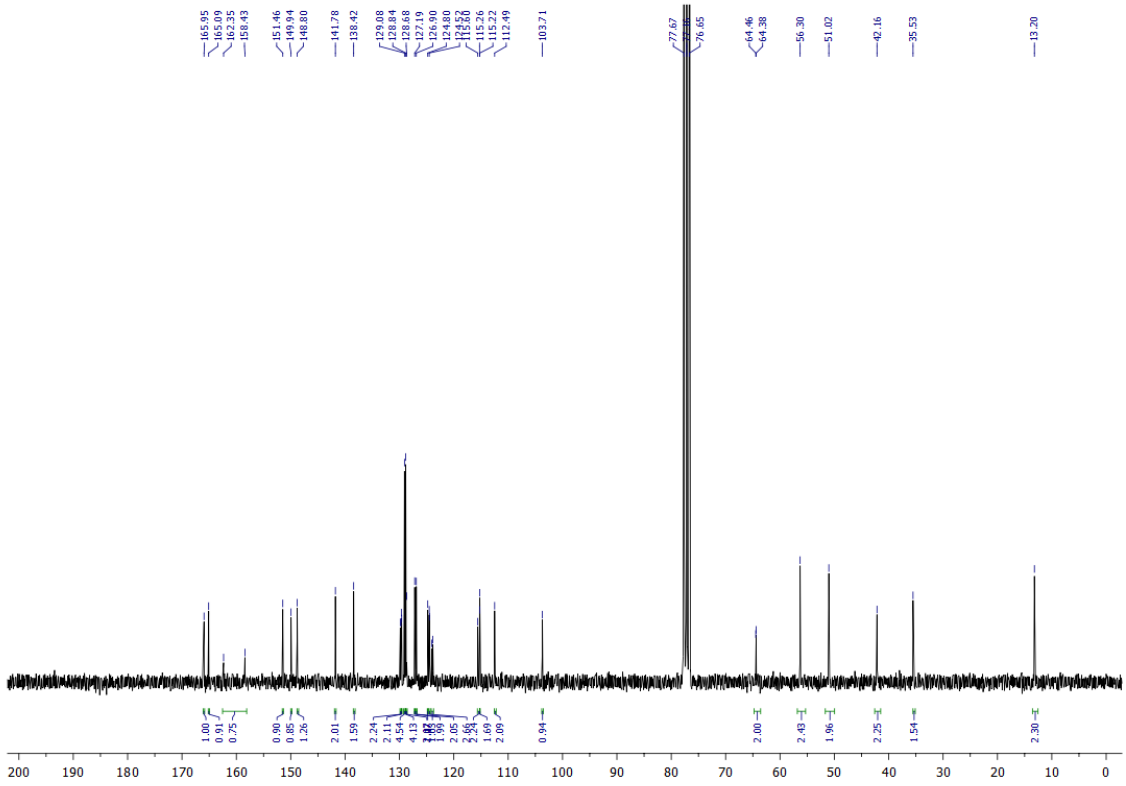

Methyl (*Z*)-4-[4-[(2-chlorobenzyl)oxy]-3-methoxybenzylidene]-2-methyl-5-oxo-1-phenethyl- 4,5-dihydro-1*H*-pyrrole-3-carboxylate (**3k**)

^{1}H NMR (250 MHz, $CDCl_3$)

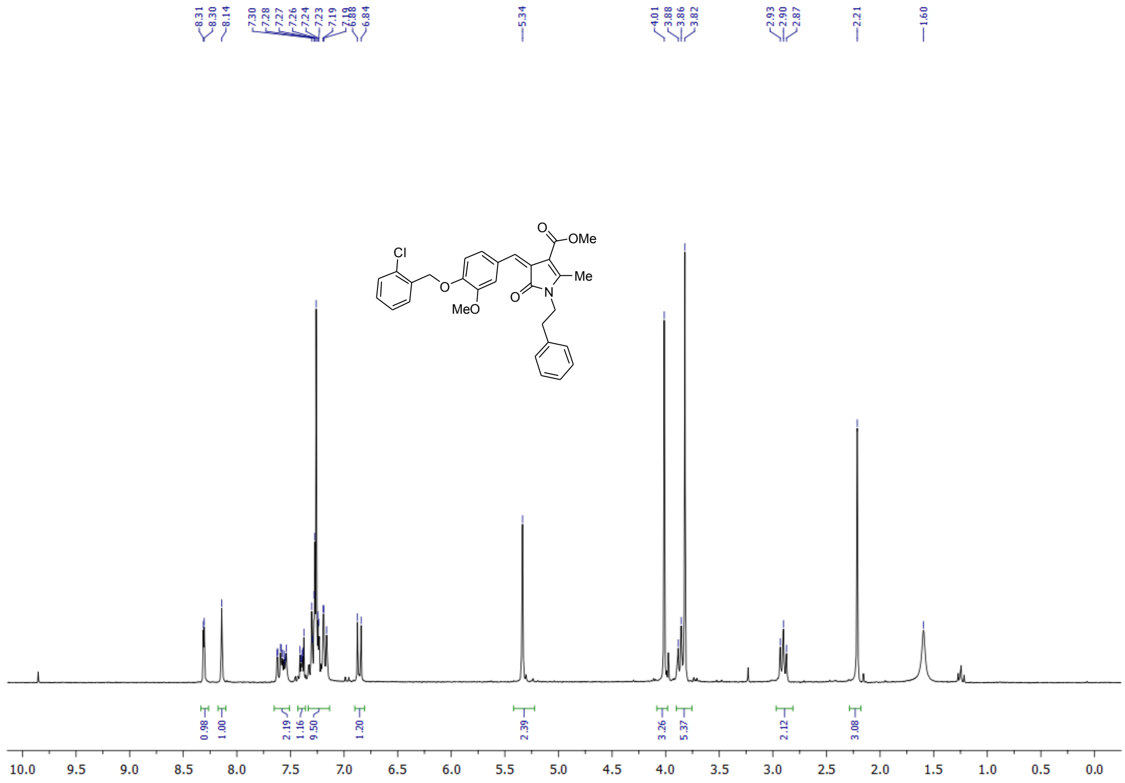

^{13}C NMR (63 MHz, $CDCl_3$)

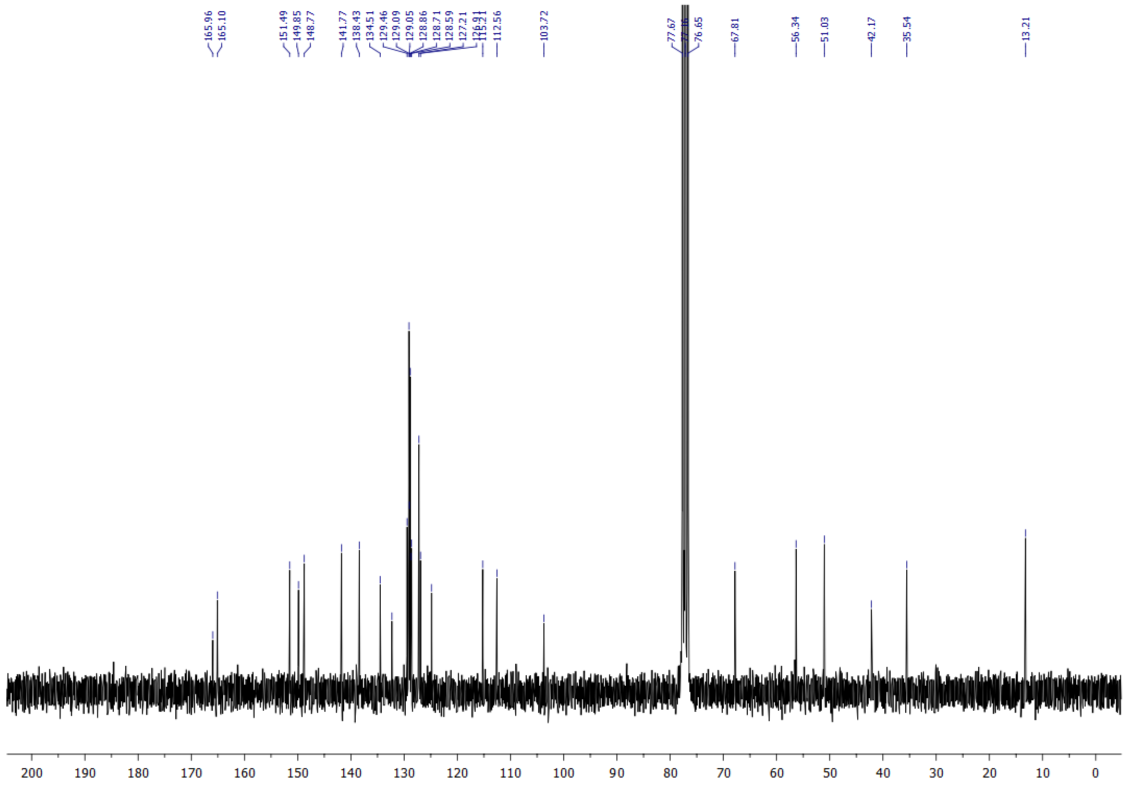

Methyl (*Z*)-4-[4-[(4-chlorobenzyl)oxy]-3-methoxybenzylidene]-2-methyl-5-oxo-1-phenethyl- 4,5-dihydro-1*H*-pyrrole-3-carboxylate (**3l**)

^{1}H NMR (250 MHz, CDCl$_3$)

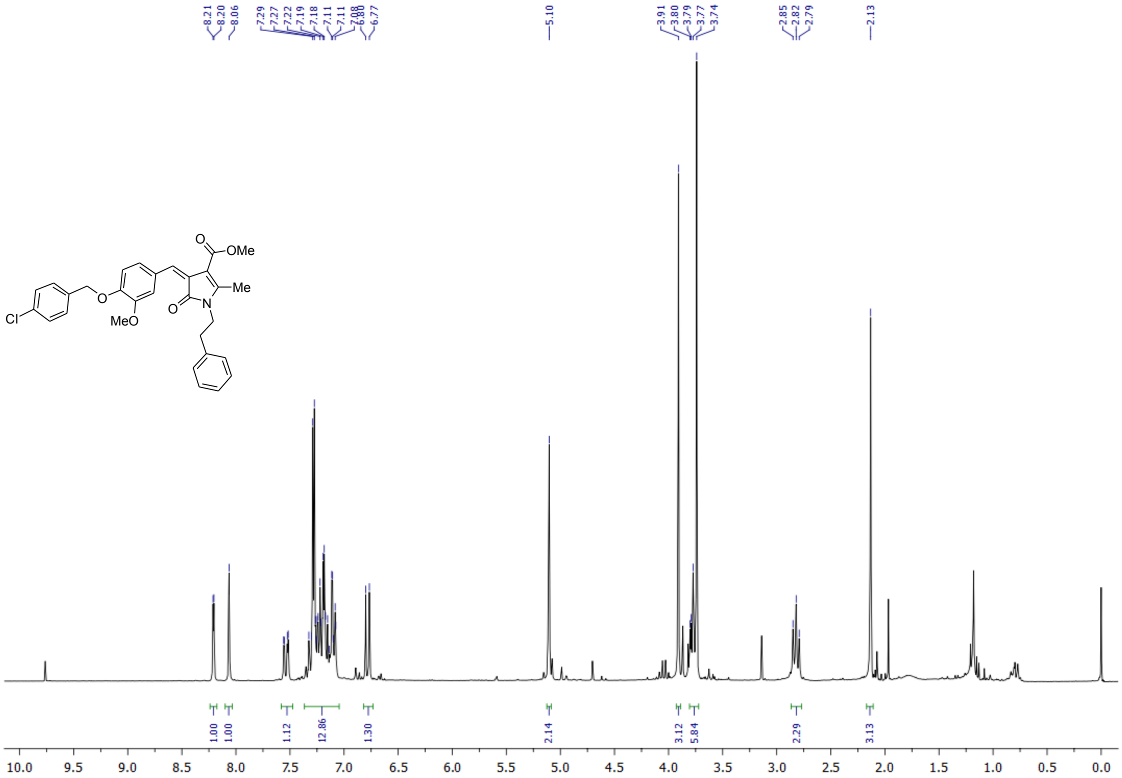

^{13}C NMR (63 MHz, CDCl$_3$)

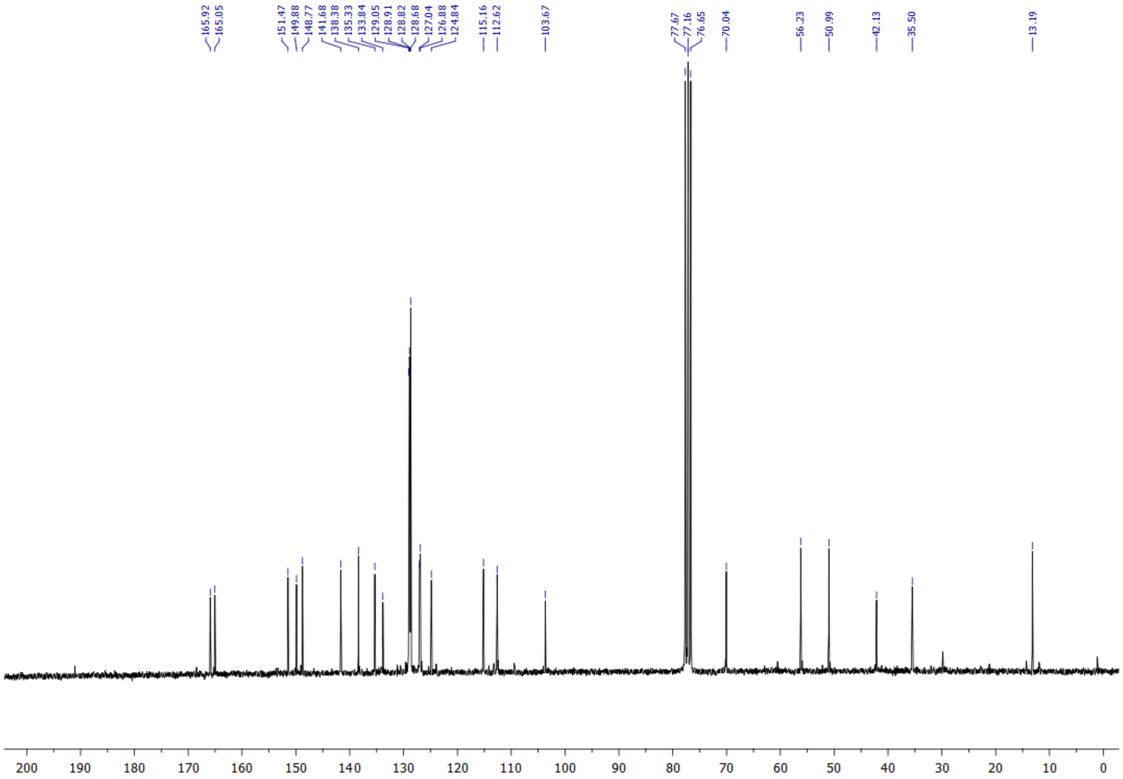

Methyl (*Z*)-4-[4-[(4-chlorobenzyl)oxy]-3-methoxybenzylidene]-1-(3,4-dimethoxyphenethyl)- 2-methyl-5-oxo-4,5-dihydro-1*H*-pyrrole-3-carboxylate (**3m**)

^{1}H NMR (250 MHz, CDCl$_3$)

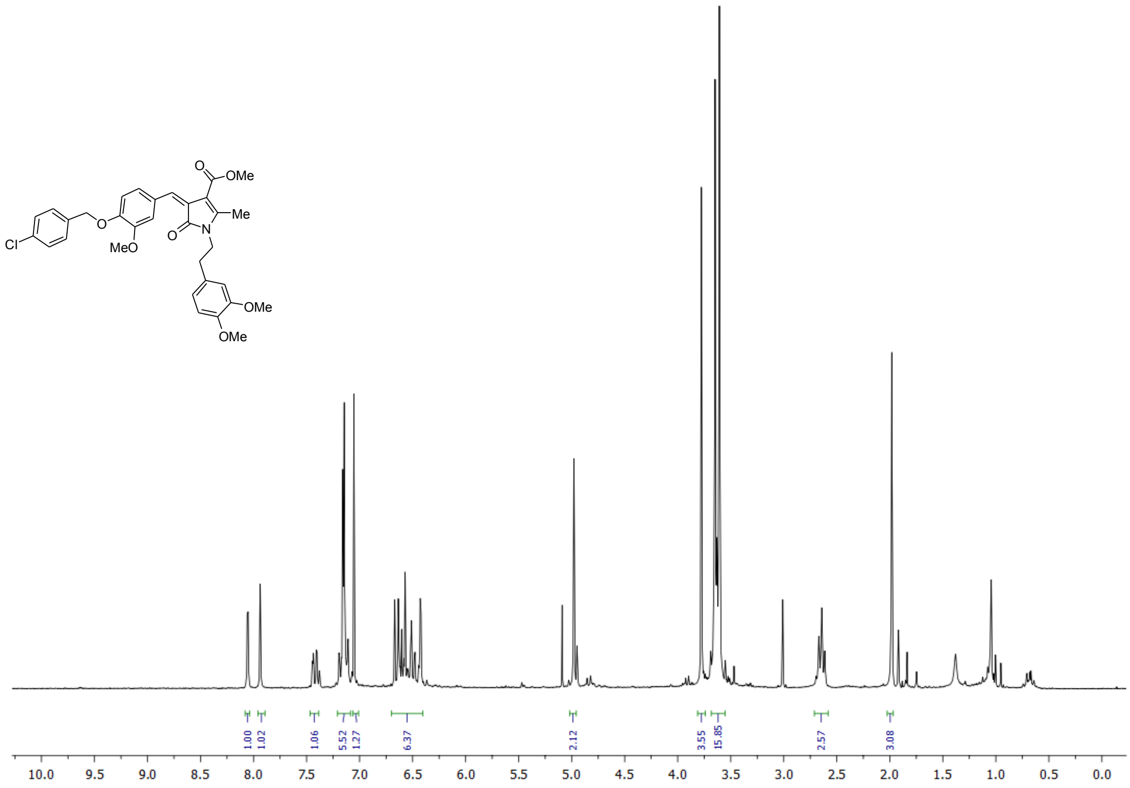

^{13}C NMR (63 MHz, CDCl$_3$)

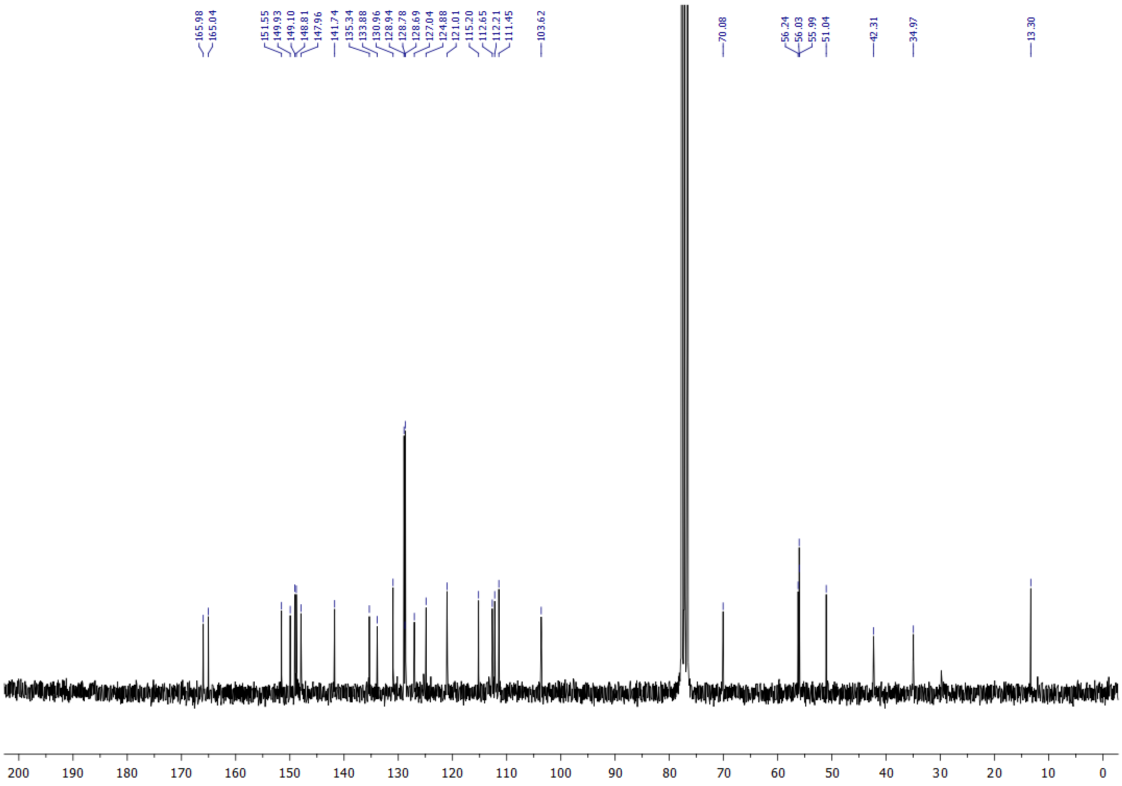

Methyl (*Z*)-4-[4-[(2-chlorobenzyl)oxy]-3-methoxybenzylidene]-1-(3,4-dimethoxyphenethyl)- 2-methyl-5-oxo-4,5-dihydro-1*H*-pyrrole-3-carboxylate (**3n**)

^{1}H NMR (250 MHz, CDCl$_3$)

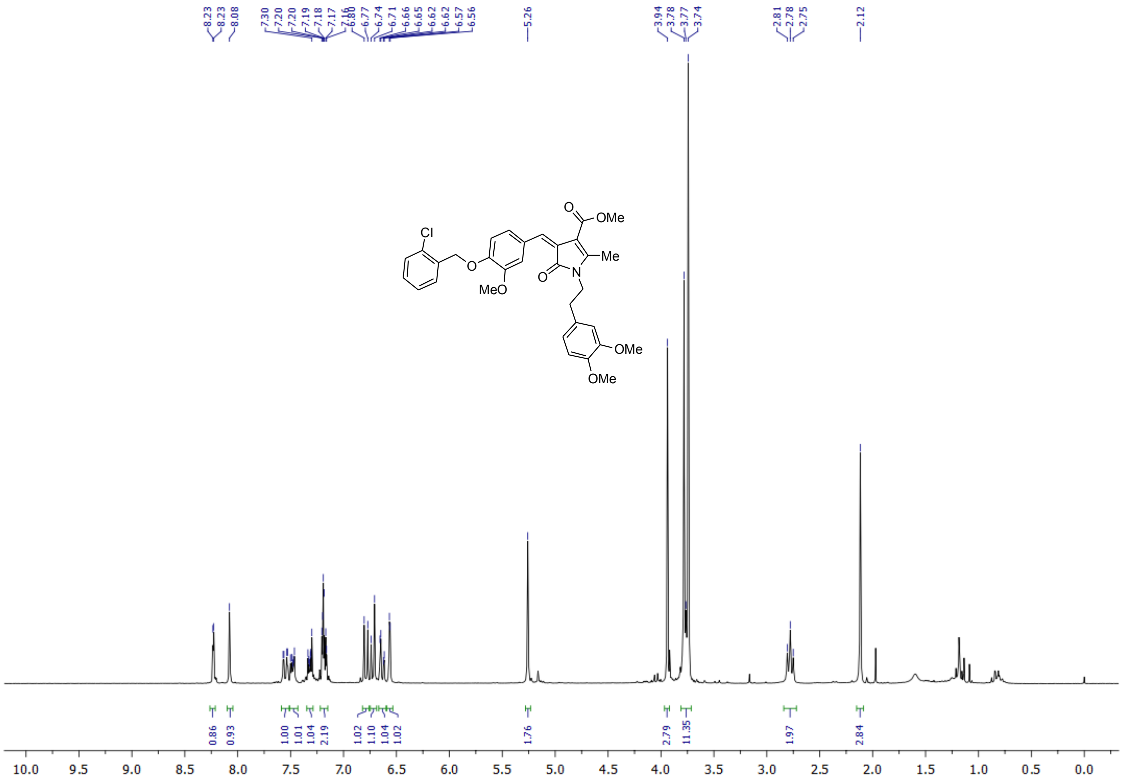

^{13}C NMR (63 MHz, CDCl$_3$)

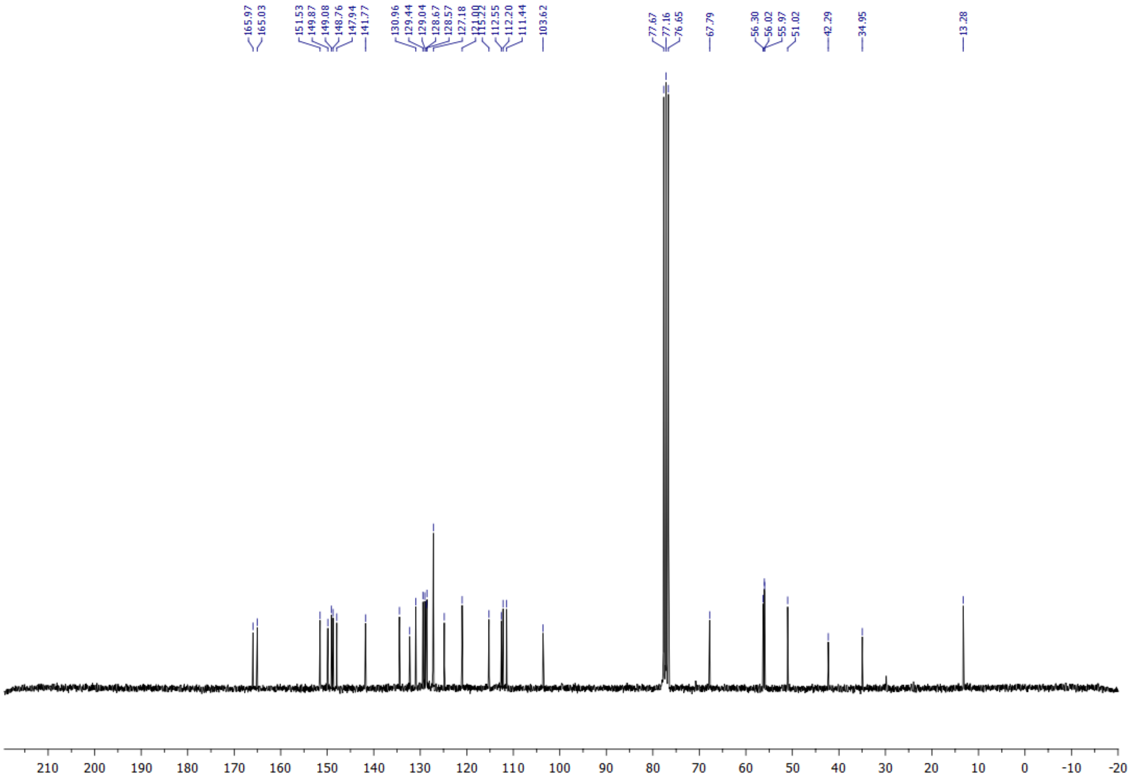

Methyl (*Z*)-4-[(1*H*-indol-3-yl)methylene]-2-methyl-5-oxo-1-phenethyl-4,5-dihydro-1*H*-pyrrole-3-carboxylate (**3o**)

^{1}H NMR (250 MHz, d_6-DMSO)

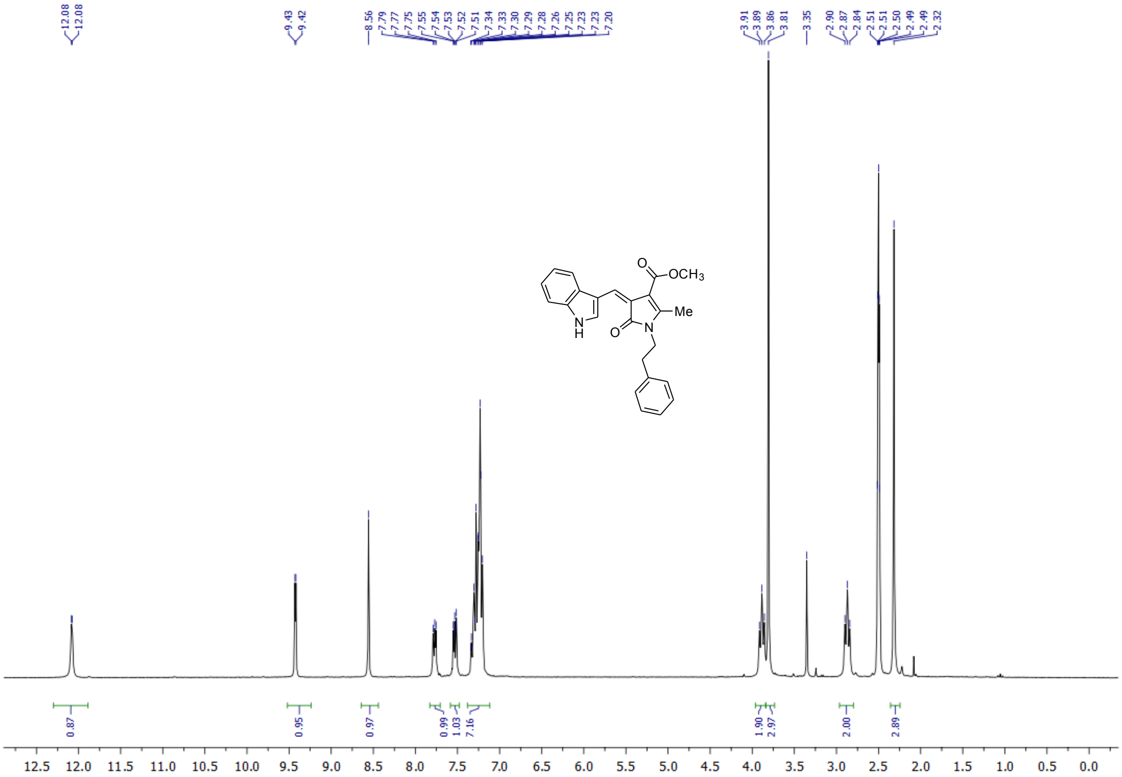

^{13}C NMR (63 MHz, d_6-DMSO)

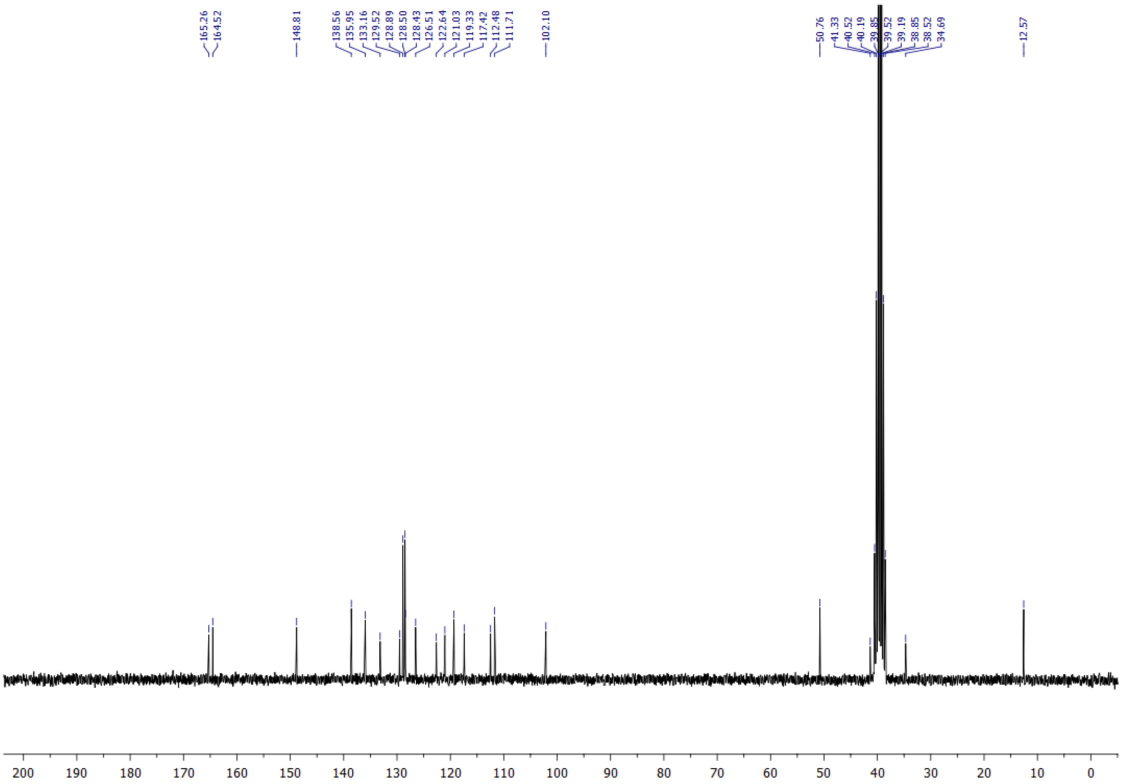

Supplement: Supplementary file 1 [file antioxidants-10-00941-s001.zip › antioxidants-1241317-supplementary.pdf]
